# Targeting lncRNAs to Overcome Cancer Therapy Resistance: Advances in RNA Therapeutics and Delivery Strategies

**DOI:** 10.3390/cancers18152497

**Published:** 2026-08-04

**Authors:** Christos Drosos, Athina Kapsi, Anthi Nikolaidou, Antonis Giakountis

**Affiliations:** Department of Biochemistry and Biotechnology, University of Thessaly, Biopolis, Mezourlo, 41500 Larissa, Greece

**Keywords:** cancer, therapeutic resistance, lncRNA, RNA therapeutics, lncRNA-targeted therapy, drug delivery systems

## Abstract

Non-coding RNAs (ncRNAs) are increasingly recognized as important regulators of cancer biology. Long non-coding RNAs (lncRNAs), defined as transcripts longer than 200 nucleotides, gain particular attention due to their cancer-specific expression patterns and functional roles in tumor progression, metastasis and therapeutic resistance. Although lncRNAs have been extensively studied as diagnostic, prognostic and predictive biomarkers, growing evidence indicates that they can also act as active mediators of therapeutic resistance, supporting their potential as therapeutic candidates. Here, we summarize how lncRNAs contribute to resistance against radiotherapy, chemotherapy, immunotherapy and targeted therapy, highlighting their molecular mechanisms. Next, we discuss RNA-based therapeutics as a strategy to target disease-relevant transcripts, focusing on antisense oligonucleotides (ASOs), small interfering RNAs (siRNAs), microRNA (miRNA) mimics and antimiRs, as well as approved RNA therapeutic agents, oncology-focused candidates in clinical development and emerging preclinical approaches directed against lncRNAs or lncRNA-controlled regulatory axes. Finally, we examine delivery platforms, including lipid-based nanovectors, extracellular vesicles, polymeric systems and other approaches designed to overcome key translational barriers, such as RNA instability, off-target effects, immune activation, renal clearance and inefficient tumor-specific delivery. By connecting lncRNA-mediated resistance mechanisms with RNA therapeutic strategies and delivery technologies, this review highlights lncRNA-directed RNA therapeutics as a promising yet developing approach in oncology.

## Simple Summary

Cancer treatment has improved substantially over recent decades, yet many patients eventually develop resistance to therapy, limiting long-term treatment success. Increasing evidence suggests that long non-coding RNAs (lncRNAs), a class of RNA molecules that do not produce proteins, play important roles in helping cancer cells survive treatment and adapt to therapeutic pressure. These discoveries have generated interest in lncRNAs as potential therapeutic targets. At the same time, recent advances in RNA medicine have demonstrated that disease-causing RNA molecules can be successfully targeted in patients. In this review, we discuss the emerging role of lncRNAs in cancer therapy resistance and examine how advances in RNA-based treatments may create new opportunities to overcome this challenge. We also highlight key obstacles that must be addressed before lncRNA-targeted therapies can be translated into clinical practice. Improved understanding of these mechanisms may ultimately contribute to more effective and durable cancer treatments.

## 1. Introduction

Cancer remains one of the leading causes of morbidity and mortality worldwide, accounting for millions of new cases and deaths each year [1,2]. Over the past decades, major advances in early detection, molecular diagnosis and therapeutic development have improved clinical management and patient outcomes [3]. Current treatment strategies include surgery, radiotherapy, chemotherapy, immunotherapy and targeted molecular therapies, either alone or in combination. Despite these advances, therapeutic resistance remains a major obstacle to durable treatment responses [4,5]. Many patients receiving anticancer therapy either fail to respond initially or eventually develop resistance during treatment, leading to tumor recurrence, disease progression and reduced therapeutic efficacy [6]. This persistent clinical challenge highlights the need to better understand the molecular mechanisms that allow cancer cells to survive treatment and to identify new strategies for overcoming resistance [7].

Only a small fraction of the human genome encodes for proteins, while most transcriptional activity originates from non-coding regions [8]. Traditionally, protein-coding messenger RNAs (mRNAs) were considered the central mediators of gene expression because they serve as templates for protein synthesis. However, over recent decades, increasing evidence has shown that non-coding RNAs (ncRNAs) are not mere transcriptional by-products, but important regulators of diverse biological processes [9]. In cancer, ncRNAs have attracted particular interest because they can display cancer-specific expression patterns and regulate pathways involved in tumor initiation, progression, metastasis and therapeutic response. Among the different ncRNA classes, long non-coding RNAs (lncRNAs) have gained particular attention [10]. LncRNAs are generally defined as transcripts longer than 200 nucleotides with limited or no protein-coding potential, although some may contain small open reading frames encoding functional micropeptides [11]. Unlike protein-coding RNAs, lncRNAs often display increased levels of context-dependent expression and can act in the nucleus or cytoplasm through interactions with DNA, RNA, proteins or chromatin-regulatory complexes [12]. Through these diverse mechanisms, lncRNAs regulate key cancer-related processes, including proliferation, metastasis, tumor microenvironment remodeling and therapeutic response [13,14].

The growing recognition of ncRNAs and lncRNAs as functional regulators of cancer has also stimulated interest in RNA-based therapeutic strategies. Unlike conventional therapies that mainly target proteins or cellular pathways at the translational level, RNA therapeutics can modulate disease-relevant transcripts directly in a sequence-specific manner [15]. Approaches such as antisense oligonucleotides (ASOs), small interfering RNAs (siRNAs), microRNA (miRNA) mimics and antimiRs have already demonstrated clinical potential in several diseases, while an increasing number of RNA-based agents are being evaluated in oncology [16]. However, their successful application in cancer depends not only on target selection, but also on effective organ- or tissue- specific delivery, as therapeutic RNAs must remain stable in circulation, reach tumor tissues, enter target cells and release their cargo intracellularly [15,17].

This review was designed to provide a mechanistic and translational overview of lncRNAs in cancer therapy resistance and their potential as candidates for RNA-based therapeutics. The literature was selected to emphasize studies that provide mechanistic insight into lncRNA biology, including subcellular localization, molecular mechanisms of action, and roles in therapeutic resistance, while also highlighting translational advances in RNA therapeutics and delivery technologies. Priority was given to seminal studies, recent developments, and representative examples across major cancer types and therapeutic modalities. Oncology-focused RNA therapeutic candidates were identified primarily through ClinicalTrials.gov and selected according to their relevance to cancer, therapeutic modality, delivery strategy, translational significance, and availability of clinical information. For delivery-related clinical examples, preference was given to clinically relevant platforms with direct oncological applicability and translational potential, particularly when their mechanisms of encapsulation, tumor targeting, controlled release, or intracellular delivery had been investigated in cancer settings. When multiple clinical trials were available for the same therapeutic agent or delivery platform, representative studies were prioritized based on oncology relevance, clinical phase, recency, trial status, and the availability of mechanistic or delivery-related information. All clinical trial statuses, regulatory approvals, discontinuation claims, routes of administration, and delivery-platform classifications were verified against ClinicalTrials.gov, regulatory agency information, and the relevant primary literature.

Although this review is not intended to be a systematic review or meta-analysis, we sought to provide a balanced overview of the field by integrating foundational and recent evidence relevant to the biological and therapeutic aspects of lncRNAs based on preclinical studies. Building on this framework, we first discuss how lncRNAs contribute to cancer therapeutic resistance through diverse molecular mechanisms. Then we broadly examine the current clinical landscape of RNA therapeutics, including approved agents and oncology-focused candidates under clinical evaluation. Building on this framework, we focus on emerging preclinical RNA-based therapeutic strategies directed against lncRNAs or lncRNA-controlled regulatory axes. Finally, we highlight delivery approaches and translational challenges that must be addressed to advance lncRNA-directed therapeutic strategies toward clinical application in oncology.

## 2. LncRNA-Mediated Mechanisms in Cancer Therapy Resistance

Recent studies investigating mechanisms of therapeutic resistance have increasingly implicated ncRNAs, especially lncRNAs, as important indicators of treatment response [18]. LncRNAs have gained considerable attention due to their high cancer- and tumor stage-specificity, properties that make them particularly promising as diagnostic, prognostic and predictive biomarkers [19,20]. However, even though many studies associate lncRNAs with therapeutic resistance, few provide direct mechanistic evidence that support their therapeutic efficacy [21]. Given this functional line of evidence is crucial, the following sections focus on lncRNAs that not only correlate with resistant phenotypes but also participate mechanistically in therapeutic escape.

### 2.1. Radioresistance

**Figure 1:**
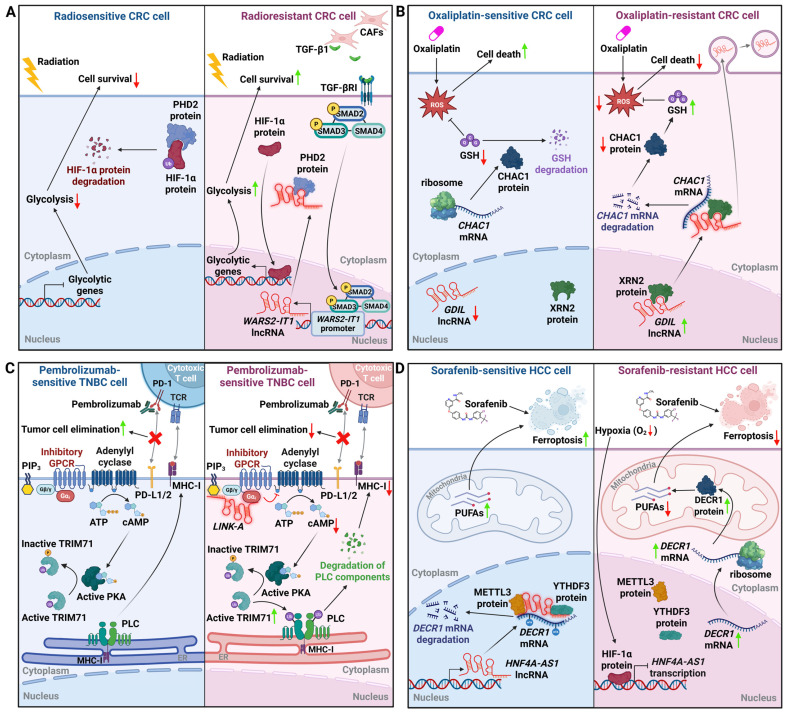
Representative mechanisms of lncRNA-mediated resistance to different cancer therapies. The cancer models shown are intended to illustrate distinct therapeutic resistance mechanisms rather than to represent a comparison of cancer-specific biology. (**A**) Radioresistance. In radiosensitive CRC cells, PHD2 promotes HIF-1α degradation, limiting glycolysis and survival after irradiation. In resistant cells, CAF-derived TGF-β signaling induces *WARS2-IT1* expression, which binds PHD2 and prevents PHD2-mediated HIF-1α degradation. Stabilized HIF-1α enhances glycolysis and promotes cell survival following irradiation. (**B**) Chemoresistance. In oxaliplatin-sensitive CRC cells, CHAC1 promotes GSH degradation, allowing oxaliplatin-induced ROS accumulation and cell death. In resistant cells, lncRNA *GDIL* promotes the translocation of XRN2 to the cytoplasm and recruits it to *CHAC1* mRNA. The resulting reduction in CHAC1 expression preserves GSH levels, limits ROS accumulation and reduces oxaliplatin-induced cytotoxicity. (**C**) Immune resistance. In pembrolizumab-sensitive TNBC cells, cAMP-PKA signaling inhibits TRIM71, preserving PLC (peptide-loading complex) components and MHC-I antigen presentation. PD-1 blockade can therefore support cytotoxic T-cell recognition and tumor cell elimination. In resistant cells, lncRNA *LINK-A* connects PIP3-associated signaling with inhibitory GPCR-Gαi signaling, suppressing adenylyl cyclase, cAMP production and PKA activity. TRIM71 consequently remains active and promotes PLC degradation, reducing MHC-I presentation and T-cell recognition. (**D**) Resistance in targeted therapy. In sorafenib-sensitive HCC cells, lncRNA *HNF4A-AS1* recruits METTL3, an m6A writer, and YTHDF3, an m6A reader, to *DECR1* mRNA, promoting m6A-dependent degradation. Reduced DECR1 expression preserves PUFAs and facilitates sorafenib-induced ferroptosis. Under hypoxia, HIF-1α represses lncRNA *HNF4A-AS1*, increasing *DECR1* mRNA expression and mitochondrial PUFA catabolism. The resulting reduction in lipid peroxidation limits ferroptosis and promotes sorafenib resistance. Abbreviations: lncRNA = long non-coding RNA, CRC = colorectal cancer, PHD2 = prolyl hydroxylase domain protein 2, HIF-1α = hypoxia-inducible factor 1 alpha, CAFs = cancer-associated fibroblasts, mRNA = messenger RNA, GSH = glutathione, ROS = reactive oxygen species, TNBC = triple negative breast cancer, PD-1 = programmed cell death protein 1, PD-L1/2 = programmed cell death ligand 1/2, TCR = T-cell receptor, PIP3 = phosphatidylinositol-(3,4,5)-triphosphate, GPCR = G-protein-coupled receptor, cAMP = cyclic AMP, PKA = protein kinase A, PLC = peptide-loading complex, MHC-I = major histocompatibility complex class I, HCC = hepatocellular carcinoma, PUFAs = polyunsaturated fatty acids. Created in BioRender. (2026) https://BioRender.com/ac3mbut.

Radiotherapy is widely used for treating multiple malignancies, primarily through its cytotoxic effects via generation of free radical species that induce double-strand breaks in DNA [22]. Although such lesions can trigger tumor cell death, cancer cells may acquire adaptive responses that limit radiation-induced damage, promoting survival and ultimately reducing therapeutic efficacy. Radioresistance may arise through several biological processes, including enhanced DNA repair, altered redox balance, activation of survival signaling, enrichment of cancer stem cells and autophagy-dependent stress adaptation. In this context, accumulating evidence indicates that lncRNAs contribute to radioresistance by regulating key molecular pathways involved in DNA damage response, oxidative stress, cell death and cellular recovery after irradiation (Figure 1, Table 1) [23].

A critical pathway implicated in the acquisition of radioresistance is autophagy, as dysregulated autophagic flux can enable tumor cells to survive radiation-induced stress. In colorectal cancer (CRC), overexpression of the lncRNA *SP100-AS1* has been shown to promote radioresistance by regulating Autophagy-related 3 (ATG3) an E2-like conjugating enzyme essential for autophagosome formation [24]. Mechanistically, *SP100-AS1* functions as a competing endogenous RNA (ceRNA) for *miR-622*, thereby preventing *miR-622*-mediated repression of *ATG3* mRNA. In parallel, *SP100-AS1* directly stabilizes ATG3 protein by inhibiting ubiquitination-dependent proteasomal degradation. Through this dual regulation at the transcript and protein level, *SP100-AS1* increases ATG3 abundance and enhances autophagic activity, ultimately supporting CRC cell survival following irradiation.

Beyond autophagy-dependent stress adaptation, lncRNAs can also regulate radioresistance by modulating the DNA damage response, a central determinant of cellular survival following irradiation. In nasopharyngeal carcinoma (NPC), lncRNA *LINC00312* functions as a negative regulator of this process and is significantly downregulated in radioresistant patient samples [25]. Specifically, *LINC00312* directly binds to the catalytic subunit of DNA-dependent protein kinase (DNA-PKcs), a key kinase involved in non-homologous end joining (NHEJ) that prevents its recruitment to the Ku70/Ku80 heterodimer, which in turn recognizes DNA double-strand breaks. This disrupts the formation of the DNA-PK complex, which acts as a scaffold for other DNA repair components. In parallel, *LINC00312* attenuates MRN-ATM-CHK2 and ATR-CHK1 signaling, further impairing DNA damage sensing and repair. Through this inhibition of DDR signaling, *LINC00312* limits DNA repair capacity and increases NPC cell sensitivity to irradiation.

Another route through which lncRNAs modulate radiosensitivity involves the regulation of ferroptosis. In NPC, lncRNA *HOTAIRM1* is upregulated in radioresistant tissues and directly interacts with FTO, a N^6^-methyladenosine (m6A) RNA demethylase [26]. This interaction enhances FTO protein stability by promoting its acetylation. Stabilized FTO removes m6A modifications from *CD44* precursor transcripts, reducing their recognition by the m6A reader YTHDC1 and shifting alternative splicing from the standard *CD44S* isoform toward the variant *CD44V* isoform. *CD44V* supports the XC cysteine glutamate reverse transport (xCT) system, preserving glutathione (GSH) synthesis and limiting lipid peroxidation, thereby suppressing ferroptosis. Consequently, *HOTAIRM1* shifts *CD44* splicing toward a ferroptosis-resistant isoform, allowing NPC cells to limit irradiation-induced lipid peroxidation and maintaining survival after radiotherapy.

Microenvironmental signaling can further shape lncRNA-mediated radioresistance. In CRC, the lncRNA *WARS2-IT1* is upregulated through cancer-associated fibroblast (CAF)-derived TGF-β signaling and promotes radioresistance by stabilizing hypoxia-inducible factor 1 alpha (HIF-1α) [27]. Mechanistically, *WARS2-IT1* interacts with prolyl hydroxylase domain protein 2 (PHD2) at the cytoplasm, disrupting the interaction between PHD2 and HIF-1α. Since PHD2 promotes HIF-1α hydroxylation and subsequent degradation, *WARS2-IT1*-mediated interference prevents this process and stabilizes HIF-1α protein. As a result, the elevated HIF-1α levels activate adaptive survival programs, including glycolytic reprogramming, thereby supporting CRC cell survival after irradiation.

### 2.2. Chemoresistance

Chemotherapy remains a cornerstone of cancer therapy. Most chemotherapeutic agents disrupt essential intracellular pathways, ultimately inducing apoptosis [28]. However, malignancies frequently develop complex resistance mechanisms that enable them to evade drug-induced cytotoxicity. In this context, lncRNAs have emerged as critical regulators of chemoresistance by modulating the expression, stability or activity of key molecular targets involved in therapeutic response (Table 2) [29].

Oxaliplatin, a platinum-based antineoplastic agent widely employed in the treatment of CRC, exerts its cytotoxic effects primarily through direct binding to DNA, as well as through interactions with RNA and cellular proteins. These interactions induce the formation of intrastrand DNA adducts, leading to cellular stress and activation of apoptotic pathways [40]. Resistance to oxaliplatin has been associated with dysregulation of several lncRNAs. For instance, in drug-sensitive cells, lncRNA-*NEF* suppresses the expression of DNA methyltransferases (DNMTs), resulting in epigenetic upregulation of DOK1 (downstream of tyrosine kinase 1), a negative regulator of the MEK/ERK signaling cascade [30]. In oxaliplatin-resistant CRC cells, lncRNA-*NEF* is significantly downregulated, relieving the inhibition of the MEK/ERK pathway and promoting tumor cell proliferation. Consequently, dysregulation of this axis enhances proliferative signaling, enabling tumor cells to sustain growth despiteoxaliplatin treatment.

Beyond epigenetic regulation, lncRNAs also contribute to oxaliplatin resistance by modulating oxidative stress responses. For example, lncRNA *GDIL* is overexpressed in resistant cells and mitigates chemotherapy-induced oxidative stress by elevating intracellular GSH levels [31]. Whereas oxaliplatin induces rapid accumulation of reactive oxygen species (ROS), elevated GSH neutralizes this oxidative burden. At the molecular level, GDIL drives the translocation of XRN2 from the nucleus to the cytoplasm, which promotes the degradation of *CHAC1* mRNA. Since *CHAC1* encodes a protein involved in GSH degradation, its suppression preserves intracellular GSH levels and enhances the ability of tumor cells to counteract oxidative stress.

Another commonly used chemotherapeutic agent in CRC is 5-fluorouracil (5-FU), which inhibits thymidylate synthase, an enzyme required for thymidine formation, thereby disrupting DNA and RNA synthesis. This results in ROS accumulation, cell cycle arrest and apoptosis [41]. Despite this cytotoxic pressure, tumor cells can adapt through lncRNA-mediated mechanisms. In CRC, lncRNA *GAS6-AS1* is overexpressed and enhances the stability of Minichromosome Maintenance Complex 3 (MCM3), a key component of the eukaryotic replicative helicase, by promoting its interaction with the RNA-binding protein PCBP1 [32]. This interaction facilitates G1/S cell cycle progression and sustains tumor proliferation, ultimately reducing the efficacy of 5-FU.

A similar mechanism has been described in gastric cancer (GC), where lncRNA *FUAT1* is upregulated in response to 5-FU-induced oxidative stress and contributes to intrinsic chemoresistance [33]. Mechanistically, *FUAT1* functions as a ceRNA for *miR-140-5p*, leading to upregulation of its downstream target *TNS4* (Tension focal adhesion 4) mRNA. Through its anti-apoptotic function, the TNS4 protein enables cancer cells to tolerate chemotherapy-induced oxidative stress without directly modulating ROS levels. Consequently, this axis suppresses ROS-mediated apoptosis and promotes tumor cell survival under therapeutic pressure.

In CRC, combination of chemotherapy regimens is commonly used, particularly including oxaliplatin and 5-FU. However, resistance remains a major clinical challenge. For instance, lncRNA-*HMG* promotes chemoresistance by suppressing ferroptosis [34]. Specifically, *HMG* acts as a scaffold that facilitates interaction between MDM2 and p53, enhancing MDM2-mediated ubiquitination and subsequent degradation of p53. This results in upregulation of downstream protein targets such as SLC7A11 and VKORC1L1, which increase antioxidant capacity, reduce lipid peroxidation and enable tumor cells to evade ferroptosis under chemotherapeutic stress.

Through an alternative mechanism, lncRNA *CACClnc* is upregulated in CRC patient samples exhibiting resistance to oxaliplatin and 5-FU [35]. At the molecular level, *CACClnc* promotes the interaction between U2AF65, a splicing factor involved in pre-mRNA processing, and YB1, an RNA-binding protein involved in gene regulation. This interaction enhances the expression of RAD51, a recombinase involved in homologous recombination and DNA repair, by preventing its aberrant alternative splicing. RAD51 also interacts with key tumor-associated proteins, including p53 and BRCA2. Ultimately, enhanced DNA repair capacity allows tumor cells to tolerate chemotherapy-induced DNA damage.

Beyond these agents, resistance mechanisms are also observed with other chemotherapeutics. Doxorubicin exerts its cytotoxic effects by inhibiting topoisomerase II and generating ROS, thus leading to DNA damage and cell death. In addition to apoptosis, doxorubicin can induce pyroptosis, a process that can be suppressed in resistant cells [42]. In BRCA, the lncRNA *STMN1P2* is significantly overexpressed in doxorubicin-resistant cells [36]. At the molecular level, *STMN1P2* inhibits doxorubicin-induced pyroptosis through interaction with hnRNPU, an RNA-binding protein, and recruitment of EZH2, an epigenetic repressor, leading to transcriptional repression of TRAF6, a key adaptor in pyroptotic signaling, and inhibition of caspase-1-dependent pathways. As a result, key pyroptotic mediators are downregulated, enabling tumor cells to evade this form of cell death and sustain proliferation under doxorubicin treatment.

Gemcitabine is a deoxycytidine analog that disrupts DNA replication through its incorporation into nascent DNA strands, resulting in premature chain termination [43]. Recent studies have implicated the lncRNA *LUCAT1* in the development of chemoresistance in bladder cancer (BC), partly through enhancement of stem cell homeostasis [37]. In this process, *LUCAT1* interacts with the RNA-binding protein IGF2BP2, which stabilizes High Mobility Group A1 (*HMGA1*) mRNA in an m6A-dependent manner. This leads to increased expression of HMGA1, a chromatin-associated transcriptional regulator. Sustained HMGA1 expression promotes stem-like properties that enhance tumor cell survival. Notably, *LUCAT1* can also be transferred via exosomes from resistant to sensitive cells, disseminating chemoresistant traits within the tumor microenvironment.

Paclitaxel exerts its cytotoxic effects by stabilizing microtubules, thereby preventing their depolymerization and inducing cell cycle arrest and apoptosis [44]. However, resistance mechanisms frequently limit its therapeutic efficacy. In triple negative breast cancer (TNBC), lncRNA *DDIT4-AS1* is upregulated, partly due to enhanced acetylation at its promoter region [38]. Mechanistically, *DDIT4-AS1* promotes autophagy by stabilizing *DDIT4* mRNA through recruitment of the RNA-binding protein AUF1. Specifically, AUF1 directly binds to *DDIT4* mRNA and prevents its degradation, thereby increasing its stability. DDIT4, an inhibitor of the mTOR signaling pathway, suppresses mTOR activity and activates autophagy. This response enables tumor cells to withstand paclitaxel-induced stress and maintain their survival.

A distinct mechanism has been described in BRCA, where poor response to paclitaxel is associated with elevated expression of lncRNA *NEAT1*, particularly in resistant cells and their exosomes [39]. Specifically, *NEAT1* functions as a ceRNA by sponging *miR-133b*, reducing its availability. As a result, *miR-133b* is unable to suppress its downstream target CXCL12, leading to increased expression of the CXCL12 protein. CXCL12, a chemokine implicated in tissue repair, stem cell homing and tumor progression, promotes tumor cell proliferation, migration and chemoresistance. Importantly, *NEAT1* can be packaged into exosomes and transferred to recipient cells, thereby propagating the resistant phenotype within the tumor microenvironment. Collectively, these findings highlight the diverse mechanisms through which lncRNAs are implicated in chemoresistance, including modulation of apoptosis, ferroptosis, autophagy, DNA repair and oxidative stress responses. Despite the heterogeneity of chemotherapeutic agents, common adaptive strategies emerge, enabling tumor cells to survive cytotoxic stress and sustain proliferation.

### 2.3. Immune Resistance

Immunotherapy has emerged as a major strategy in cancer treatment by enhancing the ability of the immune system to recognize and eliminate tumor cells. Among immunotherapeutic approaches, immune checkpoint inhibitors (ICIs) have gained clinical importance, as they inhibit immune checkpoint pathways or checkpoint molecules, such as programmed cell death protein 1 (PD-1) or programmed cell death ligand 1 (PD-L1), restoring antitumor immune activity [45]. Other strategies, including adoptive cell therapy (ACT), aim to strengthen antitumor responses through the isolation, modification and reinfusion of immune cells, such as CAR-T cells [46,47]. However, despite the clinical success of immunotherapy, resistance remains a major limitation, particularly in tumors that evade immune recognition or suppress immune activation [45]. In this context, lncRNAs have emerged as important regulators of immunotherapy response by modulating immune checkpoint signaling, antigen presentation and the immune microenvironment (Table 3) [48].

One of the most widely used immunotherapeutic agents is pembrolizumab, a PD-1 inhibitor. In TNBC, the lncRNA *LINC02544* is significantly upregulated in pembrolizumab-resistant cells [49]. *LINC02544* functions as a ceRNA by sponging *miR-497-5p*, thereby reducing its availability. In pembrolizumab-sensitive cells, *miR-497-5p* directly targets and suppresses *CAPRIN1* expression. However, increased levels of *LINC02544* relieve this repression, leading to *CAPRIN1* upregulation. CAPRIN1 has been associated with tumor progression and shows strong correlations with immune checkpoint-related pathways, suggesting a role in modulating tumor-immune interaction. Consequently, *LINC02544* overexpression promotes TNBC cell proliferation and migration, contributing to resistance after PD-1 blockade.

In addition to ceRNA-mediated regulation, alternative mechanisms have also been implicated in pembrolizumab resistance in TNBC. The lncRNA *EPIC1* is upregulated in tumor cells and contributes to immune evasion through modulation of immune signaling [50]. Mechanistically, *EPIC1* interacts with the histone methyltransferase EZH2 to repress expression of retroelements, including LINE, SINE and LTR elements, thereby reducing the accumulation of cytoplasmic double-stranded RNA (dsRNA). In pembrolizumab-sensitive cells, dsRNA is recognized by cytosolic sensors such as RIG-I and MDA5, leading to activation of type I interferon (IFN) signaling and subsequent immune activation. However, *EPIC1*-mediated suppression of dsRNA attenuates this response, resulting in reduced type I IFN signaling and decreased immune activation within the tumor microenvironment. Importantly, knockdown of *EPIC1* restores dsRNA accumulation, enhances immune cell infiltration and improves the therapeutic efficacy of pembrolizumab, highlighting its role in mediating resistance to PD-1 blockade.

Another mechanism of immune evasion in TNBC involves the lncRNA *LINK-A*. In patients who respond to pembrolizumab, *LINK-A* expression is lower and CD8+ T-cell infiltration is higher compared to non-responders, supporting its association with an immunosuppressive tumor microenvironment [51]. Mechanistically, *LINK-A* promotes crosstalk between phosphatidylinositol-(3,4,5)-triphosphate (PIP3) and inhibitory G-protein-coupled receptor (GPCR) signaling, decreasing the production of cyclic AMP (cAMP) and the activity of protein kinase A (PKA). Since PKA normally phosphorylates and restrains the E3 ubiquitin ligase TRIM71, reduced PKA activity permits TRIM71-dependent K48-linked polyubiquitination and proteasomal degradation of peptide-loading complex (PLC) components. These PLC components are required for loading antigenic peptides onto major histocompatibility complex class I (MHC-I) molecules, while their degradation impairs antigen presentation and limits CD8+ T-cell recognition. Through this mechanism, *LINK-A* promotes immune escape and reduces the effectiveness of PD-1 blockade.

### 2.4. Resistance in Targeted Therapy

Conventional cancer therapies often exhibit limited efficacy especially since heterogeneous malignancies are frequently treated with similar therapeutic strategies. Targeted molecular therapy aims to overcome this limitation by selectively inhibiting molecular alterations that drive tumor growth and progression. It includes two main types, monoclonal antibodies (mAbs), which target extracellular components, and small molecule kinase inhibitors (SMKIs), which block intracellular signaling pathways [52]. Despite their effectiveness, therapeutic resistance frequently develops, with lncRNAs emerging again as key regulators of this process by modulating pathways that influence therapeutic effectiveness and subsequent progression of the disease (Table 4).

In BRCA, trastuzumab is a widely used mAb that binds the extracellular domain of human epidermal growth factor receptor 2 (HER2), thereby inhibiting receptor dimerization and downstream signaling. However, resistance frequently develops in HER2-positive patients and recent evidence has implicated the exosomal lncRNA *LINC00969* in this process [53]. *LINC00969* is overexpressed in trastuzumab-resistant tumor cells and can be transferred to recipient cells via exosomes, facilitating dissemination of the resistant phenotype. Mechanistically, *LINC00969* acts as a molecular scaffold that promotes interaction between *HER2* mRNA and Hu antigen R (HuR), an RNA-binding protein that enhances mRNA stability. Through this interaction, *LINC00969* increases HER2 protein expression by stabilizing *HER2* mRNA without affecting its transcriptional levels. In addition, *LINC00969* overexpression is associated with increased autophagic activity, suggesting that autophagy may further support tumor cell survival under trastuzumab treatment.

Beyond mRNA-level regulation, trastuzumab resistance in HER2-positive BRCA also involves the lncRNA-driven modulation of HER2 protein stability through lncRNA *MALAT1* [54]. Specifically, tumor cells exhibit increased expression of p21-activated kinase 5 (PAK5), which phosphorylates the methyltransferase METTL14, therefore enhancing m6A modification of *MALAT1* and increasing its stability. As a result, elevated *MALAT1* levels facilitate the interaction between the deubiquitinase USP8 and nuclear HER2 (N-HER2), preventing its ubiquitin-dependent proteasomal degradation and promoting N-HER2 accumulation. The stabilized N-HER2 can act within the nucleus, supporting the transcriptional regulation of proliferation and migration, ultimately reducing sensitivity to trastuzumab.

While both trastuzumab and cetuximab target receptor-mediated signaling pathways, resistance in CRC is mediated by a distinct lncRNA-mediated mechanism involving lncRNA *UCA1* [55]. Cetuximab is a mAb that targets the epidermal growth factor receptor (EGFR), thereby inhibiting the activation of the receptor and the downstream proliferative signaling through PI3K/Akt and MAPK pathways. In cetuximab-resistant tumor cells, lncRNA *UCA1* is overexpressed and can be transferred to sensitive cells via exosomes, promoting the dissemination of the resistant phenotype. Mechanistically, *UCA1* functions as a ceRNA that sponges *miR-495*, alleviating its suppressive effect on downstream target genes. In cetuximab-sensitive cells, *miR-495* negatively regulates expression of hepatocyte growth factor (HGF) and its receptor, c-mesenchymal–epithelial transition (c-MET). However, *UCA1* overexpression leads to upregulation of both HGF and c-MET, resulting in activation of the HGF/c-MET signaling pathway. This signaling axis restores PI3K/Akt and MAPK signaling activity, effectively bypassing EGFR inhibition and sustaining tumor cell proliferation and survival despite cetuximab treatment.

Resistance to SMKIs is likewise shaped by lncRNA-mediated regulatory mechanisms. Sorafenib is a multikinase inhibitor targeting RAF kinases and receptor tyrosine kinases. It exerts its antitumor effects by suppressing tumor growth and angiogenesis as well as through induction of ferroptosis, a form of cell death driven by lipid peroxidation. In hepatocellular carcinoma (HCC), lncRNA *HNF4A-AS1*, which is involved in lipid metabolism, is downregulated in sorafenib-resistant cells [56]. At the molecular level, *HNF4A-AS1* promotes m6A modification of *DECR1* mRNA, facilitating its degradation. *DECR1* encodes an enzyme that catalyzes oxidation of polyunsaturated fatty acids (PUFAs), which serve as essential substrates for lipid peroxidation and ferroptosis. Consequently, reduced *HNF4A-AS1* expression leads to DECR1 accumulation, enhanced PUFA catabolism, and decreased lipid peroxidation, ultimately suppressing ferroptosis. This metabolic shift enables tumor cells to evade sorafenib-induced cytotoxicity and contributes to therapeutic resistance.

Another lncRNA implicated in sorafenib resistance is *LINC01056*, which is downregulated in HCC patients [57]. In sorafenib-sensitive cells, *LINC01056* interacts with peroxisome proliferator-activated receptor alpha (PPARα), a transcription factor that is predominantly localized at the cytoplasm. In contrast, in resistant cells where *LINC01056* is underexpressed, PPARα undergoes nuclear translocation. Upon entry into the nucleus, PPARα activates transcription of genes involved in fatty acid oxidation (FAO), promoting a metabolic shift from glycolysis to FAO. This reprogramming enhances ATP production, supporting tumor cell survival under sorafenib-induced stress.

Beyond metabolic adaptation, resistance to SMKIs can also arise through lncRNA-mediated bypass of cell cycle arrest, as observed for lncRNA *EILA* [58]. Palbociclib is a CDK4/6 inhibitor used in breast cancer treatment that blocks CDK4/6 activity and induces G1-phase arrest. However, activation of the cyclin E1-CDK2 complex, drives G1/S progression overcoming this arrest. In palbociclib-sensitive cells cyclin E1 is normally degraded, whereas in resistant cells its protein levels are significantly increased. Specifically, *EILA* is overexpressed and directly interacts with cyclin E1, preventing its association with the E3 ubiquitin ligase FBXW7 and inhibiting subsequent proteasomal degradation. As a result, cyclin E1 is stabilized, enabling CDK2-mediated bypass of CDK4/6 inhibition. This mechanism highlights a less common mode of lncRNA action, in which the lncRNA enhances protein stability through direct interaction with the protein rather than regulating the corresponding mRNA, allowing tumor cells to maintain cell cycle progression and survive palbociclib treatment.

Taken together, these studies establish lncRNAs as functional contributors to therapeutic resistance across radiotherapy, chemotherapy, immunotherapy and targeted molecular therapy. Their involvement in key cancer-related pathways supports the rationale for therapeutic targeting disease-relevant RNA molecules, including lncRNAs, using RNA-based approaches. In the following sections, we first summarize RNA therapeutic agents that have received regulatory approval, discuss oncology-focused RNA therapeutics currently under clinical evaluation and finally focus on the potential development of lncRNA-targeted therapeutic strategies based on preclinical evidence.

## 3. Current Landscape of RNA Therapeutics in Oncology

In recent years, RNA therapeutics have undergone rapid clinical development, with multiple RNA-based drugs advancing through clinical trials and receiving approval [59]. To date, the majority of Food and Drug Administration (FDA)- and European Medicines Agency (EMA)-approved RNA therapeutics target non-oncological indications, including rare genetic disorders, metabolic diseases, ophthalmological conditions and infectious diseases (Table 5) [60]. These successes have been largely driven by advances in oligonucleotide chemistry and delivery strategies [61,62], representing the first clinically validated examples of RNA-targeting strategies and demonstrating the feasibility of oligonucleotide chemistry, molecular target engagement, tissue-specific delivery, and regulatory approval.

In contrast, translation of RNA therapeutics to oncology has progressed more slowly. Despite strong preclinical rationale, no canonical RNA-based gene-silencing therapy for cancer has yet achieved regulatory approval, reflecting persistent challenges related to tumor-specific delivery, intratumoral heterogeneity, systemic toxicity and immune activation [59]. Nevertheless, a growing number of RNA modalities, including ASOs, small siRNAs and microRNA-based agents, are currently under clinical investigation for cancer treatment (Table 6) [84,85]. Although most agents remain in Phase I or II clinical development, their continued progression highlights sustained clinical interest in RNA-based strategies for cancer therapy. Collectively, these efforts highlight both the promise of RNA therapeutics as a new class of anticancer agents and the need to overcome key biological and translational barriers specific to cancer.

## 4. Mechanistic Classification of RNA Therapeutic Strategies in Cancer

RNA-based therapeutic strategies in cancer can be broadly classified according to their underlying mechanism of action [86,87]. One class of approaches aims to suppress oncogenic drivers through RNA-mediated silencing, thereby directly reducing expression of transcripts that promote tumor growth and therapeutic resistance. In contrast, a second class focuses on restoring tumor suppressors that are frequently disrupted in cancer, enabling the reprogramming of malignant cells toward a less aggressive and therapy-responsive phenotype.

**Figure 2:**
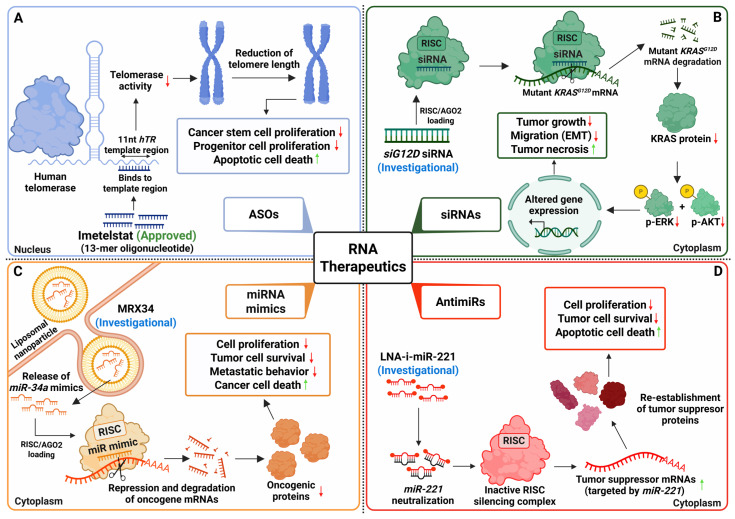
RNA therapeutics include four major categories: ASOs, siRNAs, miRNA mimics and antimiRs. (**A**) Imetelstat is an ASO-like agent approved for myelodysplastic syndromes. By inhibiting telomerase activity, it promotes telomere shortening, reduces cancer stem and progenitor cell proliferation and increases apoptotic cell death. (**B**) The *siG12D* LODER system is under clinical evaluation for pancreatic cancer. It delivers *siG12D* siRNA to promote RISC-mediated degradation of mutant *KRAS^G12D^* mRNA, suppressing KRAS signaling and reducing cancer cell proliferation and survival. (**C**) MRX34 is a liposomal nanoparticle under clinical evaluation for multiple malignancies. It delivers *miR-34a* mimics to restore tumor-suppressive activity by repressing oncogenic mRNAs, thereby inhibiting tumor growth and metastasis. (**D**) LNA-i-miR-221 is an LNA-modified antimiR under clinical evaluation for multiple malignancies. It neutralizes oncogenic *miR-221*, restoring tumor suppressor expression, thereby reducing cancer cell proliferation and promoting apoptosis. Abbreviations: hTR = human telomerase RNA, RISC = RNA-induced silencing complex, AGO2 = argonaute RISC catalytic component 2, EMT = epithelial-to-mesenchymal transition. Created in BioRender. (2026) https://BioRender.com/wc4obby.

This mechanistic framework provides a useful basis for organizing the diverse RNA therapeutics currently under investigation in oncology (Figure 2). Accordingly, in the following sections we discuss RNA-based strategies for oncogene silencing, including ASOs and siRNAs, as well as approaches aimed at restoring tumor suppressor genes using miRNA mimics and antimiRs.

### 4.1. RNA-Based Silencing of Oncogenic Drivers

RNA-based silencing strategies aim to directly suppress expression of oncogenic drivers at the transcript level [88]. Unlike conventional pharmacological approaches that target protein structure or activity, RNA-mediated silencing operates upstream of protein synthesis, enabling sequence-specific modulation of gene expression independently of protein conformation or enzymatic function [89,90]. At present, the most extensively studied RNA-based silencing approaches are ASOs and siRNAs. Both modalities have received regulatory approval from the FDA or the EMA for the treatment of several non-oncological diseases [59]. However, in the context of cancer, such agents remain largely under clinical evaluation. Among these modalities, ASOs represent the most extensively studied and clinically advanced class of RNA-based silencing therapeutics and therefore are discussed first.

#### 4.1.1. Antisense Oligonucleotides

Antisense oligonucleotides are short, single-stranded nucleic acid sequences (ssDNA or ssRNA) that are designed to bind complementary RNA targets [91]. Upon binding to their target transcripts, ASOs can regulate protein production either by promoting mRNA degradation through recruitment of endonucleases such as RNase H or by modulating pre-mRNA splicing [92]. ASOs are synthetically engineered to achieve sequence complementarity to specific targets. Furthermore, their chemical backbones incorporate a range of modifications that enhance stability and protect against nuclease-mediated degradation [93].

To date, 15 ASOs have received regulatory approval from either the FDA or the EMA (Table 5) [94]. Fomivirsen was the first approved ASO drug to receive FDA and EMA approval [95]. This ASO inhibits replication of human cytomegalovirus through binding to complementary sequences on mRNA transcribed from the major immediate-early transcriptional unit of the virus [63]. Mipomersen is a second-generation ASO developed for the treatment of homozygous familial hypercholesterolemia [96]. It acts by inhibiting apolipoprotein B-100, the principal structural component of atherogenic lipoprotein particles. Another prominent example is Nusinersen, a splice-modulating ASO approved for spinal muscular atrophy, which further illustrates the versatility of antisense-mediated gene regulation [65].

Currently, no conventional ASO targeting oncogenic mRNAs has received regulatory approval for the treatment of cancer; however, multiple ASO-based therapeutics have advanced into clinical trials (Table 6). The majority of these molecules bind to specific oncogenic mRNAs promoting their degradation [91]. BP1001 is a liposome-incorporated ASO that is currently in Phase II clinical trial **(NCT02781883)** for the treatment of acute and chronic myeloid leukemia. BP1001 binds to *GRB2* mRNA, thereby inhibiting *GRB2* expression [97]. Mechanistically, GRB2 associates with tyrosine kinases, including the Bcr-Abl chimeric oncogene found in Philadelphia chromosome-positive chronic myelogenous leukemia cells [98]. Clinical studies have reported no drug-related toxicity and have demonstrated therapeutic activity of BP1001 in these malignancies [97].

AZD9150 is another ASO that has undergone Phase II clinical trials **(NCT02983578)** for the treatment of multiple cancer types. This ASO specifically binds to *STAT3* mRNA, leading to inhibition of the STAT3 transcription factor [99]. STAT3 has proven difficult to target using traditional small molecule inhibitors; however, RNA-based therapeutics enable its direct and effective targeting at the transcript level [100]. STAT3 plays an oncogenic role in a wide range of malignancies. AZD9150 demonstrated antitumor activity by reversing resistance to chemotherapy in multiple cancer types [99]. In clinical trials, this agent was administered in combination with conventional chemotherapeutic drugs to enhance therapeutic efficacy [101,102].

AP12009 targets the mRNA of TGF-β, a key oncogenic factor that promotes tumor progression by enhancing proliferation, invasion, metastasis, angiogenesis and immune evasion [103,104]. Silencing of this pathway is achieved through downregulation of *TGF-β* mRNA. AP12009 has demonstrated antitumor activity in preclinical studies and has successfully completed a Phase IIb clinical trial **(NCT00431561)**, proving an acceptable safety profile [103].

In addition to these investigational ASO-based approaches, a recently approved therapeutic scheme highlights an alternative strategy targeting RNA components. Imetelstat is an oligonucleotide-based therapeutic approved by the FDA in 2024 for the treatment of low-risk myelodysplastic syndromes [76]. Unlike classical ASOs, it does not exert its effect through antisense-mediated RNA degradation. Instead, imetelstat is a 13-mer oligonucleotide that is covalently modified with lipid extensions and directly targets the RNA component of human telomerase (hTR), via binding with high affinity to its template region within the active site of the enzyme, thereby limiting the proliferative capacity of malignant cells [76,105,106]. Overall, these findings highlight ASOs as a promising yet clinically emerging strategy for cancer therapy and underscore the growing number of ASO-based therapeutics currently undergoing clinical evaluation.

#### 4.1.2. Small Interfering RNAs

Small interfering RNAs are double-stranded ribonucleic acid molecules that have been established as potent gene-silencing agents over the past decades. Upon cellular entry, siRNAs are incorporated into the RNA-induced silencing complex (RISC), where the sense strand is removed, resulting in the formation of the active RISC. The remaining strand directs the complex to complementary RNA sequences, typically targeting mRNAs. This interaction leads to sequence-specific cleavage of the target mRNA by the Argonaute protein, a core component of RISC, resulting in downregulation of the corresponding protein [107,108,109]. Along with ASOs, siRNAs represent one of the most extensively studied classes of RNA therapeutics [110]. Several siRNA-based therapeutics have received regulatory approval for the treatment of non-oncological diseases. In contrast, although multiple siRNAs are currently under clinical investigation, none have yet received approval for clinical use in cancer.

A major breakthrough occurred in 2018, when Patisiran received EMA approval, becoming the first siRNA-based therapeutic to gain regulatory authorization [78]. Patisiran is a double-stranded siRNA delivered to hepatocytes, where it targets the transthyretin (TTR) mRNA, leading to its degradation and subsequent reduction of circulating and tissue-deposited TTR protein [111]. This reduction resulted in clinically meaningful therapeutic benefit in patients with hereditary TTR amyloidosis.

Following the groundwork established by Patisiran, additional siRNA-based therapeutics have received regulatory approval (Table 5). Givosiran was approved by the FDA in 2019 for the treatment of acute hepatic porphyria [79]. This molecule is delivered to hepatocytes and targets the mRNA of aminolevulinate synthase 1 (ALAS1) [112]. Downregulation of this enzyme prevents accumulation of the neurotoxic metabolites δ-aminolevulinic acid and porphobilinogen, which are responsible for acute porphyria attacks.

Despite these regulatory successes beyond oncology, translation of siRNA-based therapeutics to cancer has proven more challenging, with multiple agents currently under clinical investigation. siG12D-LODER is a biodegradable, polymer-based delivery system encapsulating a KRAS-targeting siRNA developed for the treatment of advanced pancreatic cancer [113]. This agent is directly inserted into the pancreatic tumor, enabling localized and sustained release of the siRNA. The siRNA sequence is complementary to the mRNA of mutant KRAS, a member of the GTPase superfamily that is mutated in the majority of pancreatic ductal adenocarcinomas (PDAC) and is strongly associated with tumor cell proliferation and poor patient survival [114,115].

In preclinical studies, siG12D-LODER demonstrated antitumor activity by slowing pancreatic tumor growth and prolonging survival in mouse models [113]. The therapy has successfully completed Phase I/IIa clinical trial **(NCT01188785)**, where it was shown to be safe and well tolerated in patients [115]. Subsequent Phase IIb studies **(NCT01676259)** evaluated siG12D-LODER in combination with standard chemotherapy and reported encouraging therapeutic outcomes [116]. Collectively, these findings suggest that siG12D-LODER may represent a promising adjunctive strategy for the treatment of pancreatic cancer.

Another siRNA-based agent under clinical investigation is EPHARNA, which was developed to combat advanced malignant solid neoplasms. EPHARNA consists of an EphA2-targeting siRNA encapsulated in neutral 1,2-dioleoyl-sn-glycero-3-phosphatidylcholine (DOPC) nanoliposomes, enabling improved stability and tumor delivery following systemic administration [117]. The siRNA is complementary to the mRNA of EphA2, a member of the receptor tyrosine kinase family that is overexpressed in multiple cancers and has been implicated in tumor cell proliferation, survival, migration, invasion and angiogenesis [118]. In preclinical mouse models, administration of EPHARNA in combination with paclitaxel significantly reduced tumor growth compared to control treatments receiving paclitaxel and non-silencing RNA, demonstrating its therapeutic potential [117]. EPHARNA is currently being evaluated in a Phase I clinical trial **(NCT01591356)** aimed at assessing safety, tolerability and optimal dosing.

Collectively, these examples underscore the therapeutic potential of siRNA-based approaches in oncology and suggest that an increasing number of siRNA-based agents are likely to enter clinical evaluation for cancer treatment in the forthcoming years.

### 4.2. RNA-Mediated Restoration of Tumor Suppressor Genes

An alternative therapeutic strategy, distinct from oncogene silencing approaches, focuses on the RNA-mediated restoration of tumor suppressor genes and regulatory networks that are frequently disrupted in cancer. This category predominantly encompasses miRNA-based therapeutic strategies aimed at the restoration of tumor-suppressive genes or networks [119]. The principal modalities include miRNA mimics and antimiRs. miRNA mimics enable restoration of tumor suppressor miRNAs that are frequently lost or downregulated in malignant cells, thereby re-establishing repression of specific oncogenic signaling pathways [120]. In contrast, antimiRs are designed to inhibit oncogenic miRNAs, also known as oncomiRs, disrupting their tumor-promoting activity and consequently removing their suppressive effects on tumor suppressor target genes [119,120].

#### 4.2.1. miRNA Mimics

miRNA mimics are dsRNA molecules designed to resemble specific endogenous miRNAs [121,122]. This therapeutic strategy aims to restore the function of tumor suppressor miRNAs that are downregulated or lost in cancer by introducing synthetic mimics to reestablish their regulatory effects on target genes [123,124]. Similar to siRNAs, miRNA mimics have a typical length of 21-nt and are loaded into the active RISC, where they promote sequence-specific downregulation of target mRNAs [125]. Synthetic miRNA mimics can also incorporate chemical modifications to enhance stability and increase resistance to nuclease degradation [126,127].

Currently, no miRNA mimic-based therapy has received FDA or EMA approval; however, several candidates are under clinical investigation to evaluate their therapeutic potential in cancer. The first miRNA mimic to enter clinical evaluation was MRX34, a liposomal formulation designed to mimic the tumor suppressor *miR-34a* [128]. *miR-34a* is frequently downregulated or lost in multiple cancer types functioning as a key downstream effector of the p53 pathway and regulating the expression of numerous oncogenes involved in cell proliferation, survival and metastasis [129,130,131]. Restoration of *miR-34a* activity downregulates a broad network of oncogenic targets, with multiple genes reported to be affected. Preclinical studies showed that *miR-34a* mimics exerted antitumor effects in cultured cancer cell lines, including reduced proliferation, migration and invasion, particularly when combined with conventional anticancer therapies [132,133,134]. Similar therapeutic benefits were observed in mouse models [135].

MRX34 subsequently entered Phase I clinical trial **(NCT01829971)** to evaluate safety, tolerability and preliminary efficacy in patients with advanced solid tumors and hematological malignancies. Early clinical findings indicated manageable toxicity under appropriate dexamethasone premedication and provided initial evidence of antitumor activity [136]. However, a subsequent larger clinical study **(NCT02862145)** was terminated prematurely due to unexpected immune-related adverse events, including several treatment-related deaths [137]. These outcomes highlighted important safety challenges associated with systemic miRNA mimic therapy, underscoring the need for improved strategies to reduce immune activation while preserving therapeutic efficacy.

The second miRNA mimic-based therapy to enter clinical evaluation was TargomiR, a targeted delivery system consisting of EGFR-directed minicells loaded with a *miR-16* mimic [138]. *miR-16* functions as a tumor suppressor and is frequently downregulated in several malignancies, where its loss has been associated with increased tumor proliferation and decreased survival in preclinical models [139,140,141]. Restoration of *miR-16* activity suppresses growth and drug resistance of cancer cells [142]. In the Phase I clinical trial **(NCT02369198)**, the primary objectives were to evaluate safety, tolerability and optimal dosing in patients. Although the trial demonstrated an acceptable safety profile for the therapeutic scheme, the study was limited by a small patient cohort and the absence of serial tumor biopsies, preventing definitive assessment of target engagement and tumor-suppressive activity [143]. Nevertheless, the authors concluded that TargomiRs warrant further clinical investigation.

A third miRNA mimic-based therapeutic that entered clinical evaluation was INT-1B3, a lipid nanoparticle (LNP)-formulated synthetic *miR-193a-3p* mimic [144]. *miR-193a-3p* functions as a tumor suppressor in multiple cancer types, regulating genes involved in cell cycle control, apoptosis and metastasis [145]. Preclinical studies showed that INT-1B3 inhibited tumor growth and metastasis while prolonging survival in mouse models, supporting its therapeutic potential [146]. Based on these findings, INT-1B3 advanced to a Phase I/Ib clinical trial **(NCT04675996)** aimed at evaluating safety, tolerability and preliminary clinical activity. However, the clinical development program was ultimately terminated due to insufficient funding rather than safety concerns.

Together, the clinical findings from miRNA mimic-based therapies demonstrate both the therapeutic potential of restoring tumor suppressor networks and the challenges associated with safety, delivery and achievement of sustained clinical efficacy in patients.

#### 4.2.2. AntimiRs

AntimiRs are antisense oligonucleotides developed to inhibit the activity of overexpressed miRNAs [147]. By binding to their target miRNAs in a sequence-specific manner, antimiRs prevent their interaction with cognate mRNA targets, thereby disrupting miRNA-mediated gene silencing. This approach primarily targets oncogenic miRNAs that repress tumor suppressor pathways, enhance cellular proliferation and contribute to the development of chemoresistance [123,148].

Similarly to miRNA mimics, no antimiR-based therapeutic approach has received regulatory approval from either the FDA or the EMA. However, three candidates have advanced into clinical trials to further evaluate their safety and therapeutic potential. The first antimiR to enter clinical evaluation was cobomarsen (MRG-106), a synthetic oligonucleotide targeting *miR-155*, an oncogenic miRNA frequently overexpressed in hematological malignancies [149,150]. *miR-155* regulates multiple tumor suppressor pathways, including TP53INP1, a p53-inducible factor involved in DNA damage response and apoptosis [151,152]. Cobomarsen showed a manageable safety profile and low toxicity in a Phase I clinical trial **(NCT02580552)** completed in 2020 [153]. A subsequent Phase II study **(NCT03837457)** was designed to evaluate its efficacy in patients with cutaneous T-cell lymphoma (CTCL) of the mycosis fungoides subtype, in comparison to the histone deacetylase inhibitor vorinostat. Although cobomarsen is compatible with a less frequent dosing therapeutic scheme, it required intravenous administration, unlike the oral delivery of vorinostat. The trial was ultimately terminated for business-related reasons, while a follow-up Phase II study **(NCT03713320)** was discontinued due to insufficient patient recruitment.

The second antimiR under clinical investigation is LNA-i-miR-221, a 13-mer locked nucleic acid (LNA)-modified oligonucleotide with a fully phosphorothioate backbone targeting *miR-221*, an oncogenic miRNA implicated in tumor growth and survival across multiple malignancies [154,155]. Preclinical studies demonstrated significant antitumor activity and favorable toxicokinetic profiles, supporting its progression to clinical evaluation [156]. In a Phase I study **(NCT04811898)**, LNA-i-miR-221 was well tolerated, with no dose-limiting toxicities observed, and demonstrated preliminary signs of clinical activity, including disease stabilization and partial response in a subset of patients [157]. Pharmacodynamic analyses indicated target engagement, evidenced by downregulation of *miR-221* and upregulation of its tumor suppressor targets. These findings support further clinical investigation of this therapeutic strategy, although it has not yet progressed to Phase II evaluation.

## 5. Emerging lncRNAs as Candidates for Therapeutic Success in Cancer: Preclinical Evidence

Although currently approved RNA therapeutics and cancer clinical trials are mainly focused on protein-coding transcripts or miRNA modulation, lncRNAs represent an alternative, emerging class of therapeutic candidates (Table 7). This is particularly relevant in oncology given the multiple examples of lncRNAs involved in therapy resistance, tumor progression and metastatic behavior. Despite the lack of extensive clinical evaluation, a growing body of preclinical and early translational evidence supports the potential of lncRNAs as biomarkers for predicting disease relapse or potential candidates for therapeutic targeting in cancer.

In platinum-resistant NSCLC, *lncRNA16* was identified as a regulator of chemoresistance through suppression of pyroptosis. Previous work showed that *lncRNA16* is upregulated in platinum-resistant NSCLC tissues and is mainly localized at the cytoplasm, where it functions as a ceRNA by sponging miRNA1827 [163]. Through this interaction, *lncRNA16* regulates downstream genes that ultimately inhibit pyroptosis, thereby supporting platinum resistance. To counteract this axis, a miRNA mimic strategy, including a *miRNA1827* agomir, was used to restore *miRNA1827* activity. In platinum-resistant NSCLC cell lines, treatment with the *miRNA1827* agomir reduced colony formation and cell viability, with stronger effects observed in combination with cisplatin. Consistently, in platinum-resistant cell line-derived xenograft (CDX) models, *miRNA1827* agomir suppressed tumor growth, while combination treatment with cisplatin further enhanced tumor inhibition without affecting mouse body weight. This chemosensitizing effect was also validated in platinum-resistant patient-derived xenograft (PDX) models. In these PDXs, cisplatin alone had minimal effect, whereas *miRNA1827* agomir alone or in combination with cisplatin significantly inhibited tumor growth [158]. These findings suggest that restoring *miRNA1827* activity may overcome *lncRNA16*-mediated platinum resistance and provide a potential chemosensitizing strategy in NSCLC.

In head and neck squamous cell carcinoma (HNSCC), *AC104041.1* has been identified as an oncogenic lncRNA that promotes tumor growth and metastasis. Mechanistically, this lncRNA acts as a ceRNA for *miR-6817-3p*, thereby reducing *miR-6817-3p*-mediated repression of Wnt2B and maintaining activation of the Wnt/β-catenin pathway. To evaluate its tumor-suppressing potential, LNA-modified ASOs targeting *AC104041.1* were used [159]. *AC104041.1* inhibition reduced HNSCC cell viability and migration in vitro, suppressing tumor growth in PDX models. Notably, combination treatment with salinomycin, a Wnt signaling inhibitor, further enhanced the antitumor effect of *AC104041.1* ASOs, leading to stronger inhibition of cell viability, migration and tumor growth than the effect induced by either treatment alone. These results suggest that ASO-mediated targeting of *AC104041.1*, particularly in combination with Wnt pathway inhibition, may enhance conventional therapeutic strategy for HNSCC.

*LOC644656* has also been proposed as a potential, preclinical therapeutic candidate in tumors such as liver hepatocellular carcinoma (LIHC) and kidney renal clear cell carcinoma (KIRC) [160]. In cancer cell models treated with 5-FU, *LOC644656* expression reduced genotoxic stress-induced phosphorylation of DNA-PKcs and activation of γH2AX, indicating suppression of DNA damage response signaling. In contrast, ASO-mediated knockdown of *LOC644656* enhanced DNA-PKcs signaling and p53 activation, while increasing cellular sensitivity to 5-FU. Consistent with this, 3D culture and CDX experiments showed that *LOC644656* expression promoted resistance to chemotherapy-induced genotoxic stress. These results indicate that targeting this lncRNA with ASOs may help to overcome chemoresistance by restoring DNA damage response activation in selected cancers.

In GC, *MALAT1* has been implicated in tumor progression and chemoresistance through its transfer from M2-polarized tumor-associated macrophages (TAMs) via exosomes [161]. Exosomal *MALAT1* promoted tumor growth, metastasis and resistance to oxaliplatin, partly by enhancing glycolysis. In vivo, M2 macrophage-derived exosomes increased tumor growth and metastatic burden, whereas depletion of *MALAT1* from these exosomes attenuated these effects and improved the therapeutic efficacy of oxaliplatin. To further explore in vivo therapeutic potential, engineered exosomes were loaded with *MALAT1* siRNA, allowing simultaneous targeting both GC cells and M2 TAMs. This approach efficiently reduced *MALAT1* expression in both cell populations, suppressed tumor growth and further enhanced the antitumor effect of oxaliplatin in mouse xenograft models. These findings suggest that exosome-mediated delivery of *MALAT1* siRNA may represent a promising strategy to disrupt tumor-microenvironment crosstalk and improve chemotherapy response in GC.

In HCC, *PURPL* has been proposed as another lncRNA with therapeutic relevance. *PURPL* is upregulated in liver cancer cells and appears to support tumor cell survival. To assess its functional role, LNA-modified gapmer ASOs targeting *PURPL* were designed. This ASO-mediated *PURPL* knockdown strategy reduced proliferation in liver cell lines and promoted apoptosis, indicating that *PURPL* may act as a pro-survival lncRNA in HCC. Importantly, *PURPL* depletion also sensitized liver cells to doxorubicin, suggesting that targeting *PURPL* may help reverse doxorubicin, resistance and improve chemotherapy response in HCC [162]. Taken together, these preclinical studies highlight lncRNAs as promising therapeutic candidates that enhance therapeutic response, or act as biomarkers for predicting therapeutic resistance. Importantly, selection of an RNA therapeutic modality cannot be generalized across all lncRNAs but should instead be guided by the biological properties of the target transcript, including its subcellular localization, mechanism of action, genomic context, and tumor specificity (discussed in detail in Section 7 Limitations and Challenges). As such, the need for detailed mechanistic characterization of cancer-associated lncRNAs is essential. Understanding how individual lncRNAs regulate tumor progression, therapeutic response, and resistance pathways is critical for predicting the biological consequences of their inhibition, selecting the most appropriate RNA-targeting strategy, and evaluating their suitability for safe and effective clinical translation.

## 6. Delivery Strategies and Considerations for RNA-Based Therapeutics in Oncology

Despite their therapeutic potential, RNA-based cancer therapies, including those targeting lncRNAs, face several limitations that hinder their clinical application. A primary concern is the risk of off-target effects, as partial sequence complementarity may lead to unintended gene regulation and disruption of normal cellular pathways [59,164]. While naked RNA molecules demonstrate promising in vitro efficacy, their systemic application remains limited by major in vivo bioavailability challenges, including rapid nuclease-mediated degradation, renal clearance, limited cellular uptake and potential immune activation [62,165,166]. In particular, RNA-based therapies may be recognized by innate immune receptors, triggering inflammatory responses and potential systemic toxicity [167,168,169].

Among these challenges, inefficient and non-specific organ delivery remains a major barrier, as it limits tumor accumulation, cellular uptake and intracellular release [170]. Because many RNA therapeutic agents share similar physicochemical properties, delivery platforms optimized for one RNA modality may often be adapted to others, although each target tissue and disease context still requires specific optimization [171,172]. In oncology, this challenge is further complicated by the heterogeneity of the tumor microenvironment, abnormal tissue architecture and variable receptor expression between malignant and healthy cells [173,174,175]. Therefore, effective delivery systems should (i) protect their RNA cargo during circulation, (ii) promote tumor accumulation, (iii) facilitate uptake by target cells and (iv) enable endosomal escape to achieve intracellular activity. Addressing these delivery-related limitations is essential for improving the safety and efficacy of RNA therapeutics. For this reason, potential strategies for delivery of therapeutic agents are discussed in the following sections.

### 6.1. Lipid-Based Nanovectors

#### 6.1.1. Lipid Nanoparticles

LNPs are the most commonly used carriers of nucleic acids as they have successfully passed preclinical and clinical phases [176,177]. They refer to small lipid nanoparticles around 100 nm in diameter consisting of a cationic ionizable lipid and other helper lipids, such as polyethylene glycol-conjugated lipid (PEGylated lipid), cholesterol, phospholipids, etc. The composition and type of lipids can affect different properties of the LNPs, as they can contribute to their stability, biodistribution, and endosomal escape [176]. Due to their lipophilic nature, LNPs can go through the cytoplasmic membrane, and given their positive charge, they can interact with negatively charged RNA molecules, protecting the latter from nucleases and facilitating their intracellular delivery.

To enter the cell, LNPs need to interact with the cell membrane. Their internalization occurs through clathrin-mediated endocytosis or micropinocytosis. The next step is to overcome the pH degradation among various vesicular structures (early endosomes pH~6.3, late endosomes pH~5.5, lysosomes pH < 5) [178]. RNA molecules must escape from the LNPs before fusing with lysosomes to prevent degradation. To achieve this, ionizable lipids were designed, since entering the acidic pH of endosomes, these modified molecules are positively charged and subsequently interact with negatively charged lipids of the endosomal membrane. This interaction fuses the membranes and releases the RNA into the cytosol of the target cells.

**Figure 3:**
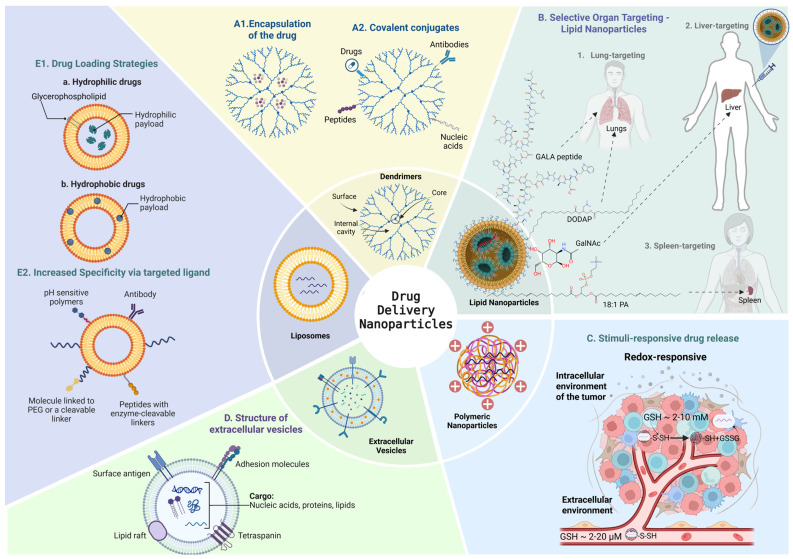
Data visualization of the five main nanoparticles used for drug delivery. (**A**) Dendrimers are a drug delivery platform with a distinctive design. They consist of three main parts: core, internal cavity, and the surface. (**A1**) Drug delivery can be accomplished by either encapsulating the drug in their inner cavity or (**A2**) by covalently conjugating it in their surface. (**B**) Lipid nanoparticles (LNPs) can transfer their cargo to specific organs with a technique called selective organ targeting. When LNPs are conjugated with GALA peptide (a sequence comprising glutamic acid, alanine, leucine and alanine) or 1,2-dioleoyl-3-dimethylammonium-propane (DODAP), their cargo can effectively reach the lungs. Were LNPs to be intravenously administered, they would end up in the liver due to the numerous apolipoprotein E (ApoE) receptors that take up LNPs. Another technique to accomplish liver targeting is by adding N-acetylgalactosamine (GalNAc) to the surface of LNPs which is the ligand of Asialoglycoprotein Receptors (ASGRs) that are mostly expressed in the hepatocytes. 18:1 phosphatidic acid (18:1 PA) is a sort molecule that drives LNPs straight to the spleen. (**C**) A great way for the controlled release of the drug is the incorporation of stimulus-responsive mechanisms. A representative example is redox-responsive release. The GSH levels in the extracellular environment range from 2–20 μM, whereas in the tumor site they range from 2–10 mM. Such high levels of GSH enable the break of the disulfide link with which polymeric nanoparticles are conjugated, leading to the upcoming release of RNA to the site of the tumor. (**D**) Extracellular vesicles can carry different types of cargoes. For the targeting to be specific, surface antigens, adhesion molecules, and lipid crafts are incorporated into the extracellular membranes of EVs. (**E1**) Liposomes are capable of carrying both hydrophilic and hydrophobic drugs, with hydrophilic ones enclosed in the nucleus and hydrophobic ones enclosed in the lipid bilayer (**E2**). Their specificity can be further enhanced by incorporating local stimuli to trigger the release of drug or antibodies. Created in BioRender. (2026) https://BioRender.com/8xxi2dg.

**Figure 4:**
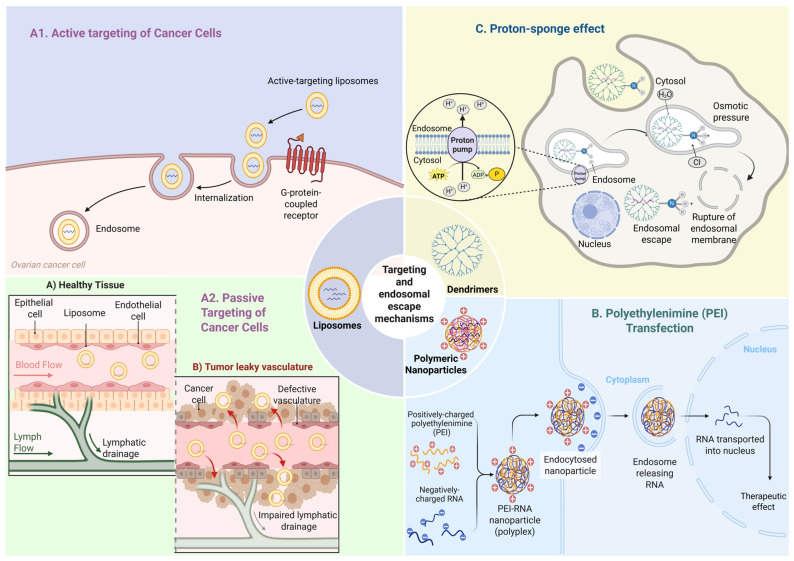
Drug delivery nanoparticles mechanisms of targeting and endosomal escape (**A1**) Passive targeting happens through the leaky vesicles of the tumor. Though these “holes” liposomes manage to reach their targeting site in the tumor, whereas (**A2**) active targeting relies on receptor-specific ligands placed on the surface of the liposome and the targeting cell. (**B**) Due to the lack of a cationic moiety, solid nanoparticles need to incorporate cationic motifs like polyethylenimine (PEI) into their structure so as to encapsulate negatively charged RNA and lead it inside the cell to reach its therapeutic effect. (**C**) Dendrimers use a technique called proton-sponge effect to make endosomal escape possible. Dendrimers containing amino groups can enter the cell via endocytosis. In the acidic environment of the endosome, they become protonated, thereby gaining a high buffering capacity. Acidification occurs due to the activity of an ATPase enzyme that transports protons from the cytosol to the surface of the dendrimer. In response to that, chloride ions enter the endosome to counteract the positive charge. This leads to the swelling of the endosome and eventually to the rupture of its membrane. Finally, dendrimers are released into the cytosol to perform their function. Created in BioRender. (2026) https://BioRender.com/2arh777.

The efficiency of LNPs for RNA molecule delivery depends on the composition of helper lipids and the circumstances under which lipids and RNA will be mixed (temperature, RNA–lipid ratio) [179]. LNPs are the most preferred delivery system in developing mRNA vaccines, siRNA, and ASO therapies [180]. A comprehensive summary of the LNPs’ drug delivery system is shown in Figure 3 and Figure 4. and Drug delivery strategies are further summarised in Table 8a,b separately for RNA silencing-based therapeutics and miRNA-based cancer immunotherapies. Although these clinical RNA platforms have not yet been applied extensively to lncRNA targets, they establish the delivery and pharmacological principles that may inform future lncRNA-directed approaches, including selection of delivery vehicles according to RNA localization, target tissue, and mechanism of action.

Asialoglycoprotein receptor (ASGR) is the most typical target when aiming to deliver a drug to the liver, as it is specifically expressed in hepatocytes [181]. The most commonly used ligand for liver-specific applications is N-Acetylgalactosamine (GalNAc) [182]. However, delivery to other organs beyond the liver has proven challenging. One main disadvantage of plain LNPs is that when delivered intravenously, they tend to be taken up by apolipoprotein E (ApoE). Given the abundance of ApoE receptors in the liver, plain LNPs or ionizable cationic lipids are restricted in this organ, thus limiting their broad therapeutic use [183]. To overcome this barrier, scientists generated selective organ-targeting (SORT) lipids. When using the cationic lipid 1,2-dioleoyl-3-dimethylammonium-propane (DOTAP) as the SORT molecule, they found that SORT-LNPs could effectively target the lungs. In general, permanent cationic lipids containing quaternary ammonium headgroups lead to lung targeting [183]. Applying GALA peptide, a synthetic amphiphilic peptide that can target the asialoglycosidic chain on lung endothelial cells, can also be a good alternative for targeting the lungs [184].

Importantly, when scientists used the negatively charged 1,2-dioleoyl-sn-glycero-3-phosphate (18PA) as the SORT molecule, SORT-LNPs targeting was re-directed to the spleen. The mechanism explaining the selective targeting is that SORT lipids can be recognized by different serum proteins that bind to them and subsequently interact with homologous receptors, delivering the LNP to specific organs [183]. Another factor that could play a crucial role in SORT delivery is the pKa of the vehicle. LNPs targeting the liver have an apparent ~pKa in the range of 6–7. However, in the lung, the apparent pKa was greater than 9, whereas for the spleen, the apparent pKa was in the range of 2–6 [183].

Emerging evidence from clinical trials supports the potential of these delivery systems. A great example is autogene cevumeran **(NCT05968326)**, which is evaluated in a Phase II clinical trial to treat resected PDAC in combination with atezolizumab and mfolfirinox. This drug contains two 5′-capped, single-stranded, uridine-based mRNA molecules that encode up to 20 neoantigens, connected via glycine- and serine-rich linkers, and encapsulated in an RNA-lipoplex formulation. The RNA backbone is designed to optimize the translational efficacy of the coding sequence in human dendritic cells and for enhanced antigen presentation on HLA class I and II molecules. The primary vector contains a secretory signal peptide and the HLA class I trafficking domain (MITD) sequences. The RNA-LPX is administered intravenously to target the dendritic cells residing in lymphoid compartments, and combining antigen delivery with the stimulation of Toll-like receptor-mediated expansion of antigen-specific T-cells. RNA-LPX was shown to engage TLR7/8 receptor-mediated inflammatory cytokine production and to induce strong IL-1β secretion of myeloid cells without causing immune activation [185]. Furthermore, LNPs can also be internalized by non-antigen-presenting cells (APCs) at the site of administration. Somatic cells are also capable of mRNA transfection, thus enhancing immunity by transferring antigens to APCs.

Another notable example is ALN-VSP02, which is in Phase I clinical trial **(NCT01158079)** and **(NCT00882180)**, aiming to treat advanced solid tumors with hepatocellular involvement. It contains two siRNAs that target (i) the vascular endothelial growth factor VEGF-A to inhibit angiogenesis and (ii) the kinesin spindle protein gene to achieve cell cycle arrest, which are chemically modified so that they reduce immune activation [186]. The delivery nanoparticle has a diameter of 80–100 nm and is uncharged with a pH set at 7.4 [186]. This complex primarily targets both the liver and the spleen following parenteral administration due to the cancerous leaky microvasculature. All patients were premedicated with a combination of drugs to reduce the risk of infusion-related reactions observed with the administration of other liposomal products [186]. After treating thirty-one patients, it was observed that all tolerated the drug, which in turn induced a tumor-suppressive effect in compliance with an anti-VEGF image [187].

Another interesting application is the use of OGX-427 (**NCT01681433)**, a drug that is in clinical Phase II and focuses on treating castration-resistant prostate cancer. It targets heat shock protein 27 (Hsp27). In this type of cancer, Hsp27 acts as a shuttle to transport the activated androgen receptor (AR) into the nucleus to function as a transcription factor. OGX-427 is a modified ASO that inhibits HPS27 expression. The efficacy of this method relies on targeting the upstream protein of the receptor and not the receptor itself, as it is prone to mutations [188].

#### 6.1.2. Liposomes

Liposomes are nanocarriers composed of naturally derived phospholipids; thus, they are biocompatible, biodegradable, non-immunogenic, and non-toxic to the organism [189]. Liposomes consist of a self-assembled bilayer of glycerophospholipids with a hydrophobic and a hydrophilic area that encloses an aqueous nucleus, mimicking the cell membrane, thus allowing cellular uptake of drugs [190]. The organized structure of liposomes allows their loading with different kinds of drugs, with hydrophilic ones enclosed in the nucleus and hydrophobic ones enclosed in the lipid bilayer [191]. Their broad application is based on their ability to protect their cargo against endonucleases, preventing fast plasma clearance in combination with their compatibility for cost-effective and large-scale industrial production. In addition, their various physicochemical characteristics are very appealing in designing drug delivery vectors [190]. Another property that plays a vital role in organ delivery is their elasticity, as it can influence circulation time and cellular uptake. Liposomes with lower elasticity interact with cells via the clathrin-mediated pathway, whereas more elastic liposomes rely on micropinocytosis. Therefore, the level of liposome, elastic design, along with their surface charge and composition, affect biodistribution and subsequent tumor accumulation [192,193].

Different types of liposomes, such as cationic (DOTAP & DOPE), neutral (DOPC), ligand-targeting, and polymer-coated liposomes exist [189]. Their organ-targeting properties can be engineered as either passive or active. Passive targeting relies on the leaky tumor vasculature, while active targeting is achieved through receptor-specific ligands on the surface of the liposome and the targeting cell [190,194]. A widely used ligand is folate; folate receptors are overexpressed in ovarian cancers. In this way, the liposome is internalized and creates endosomes through which the load is released at the target site [189].

Liposome efficiency can also be improved by integrating local stimuli to trigger the release of the drug, by taking advantage of the tumor microenvironment’s special characteristics, such as the low pH, the increased temperature, and the overexpression of specific enzymes (matrix metalloproteinases (MMPs) and phospholipase A2) [193]. To achieve this, thermosensitive lipids with a specific transition temperature from lipid to liquid phase are included during the formulation of liposomes, or alternatively, pH-sensitive polymers can be added to their core structure [193]. Furthermore, enzyme-sensitive liposomes have been constructed facilitating release of their load in response to enzymes [193]. Liposomes escape the endosome via membrane destabilization due to the direct contact between their lipid bilayer and the endosomal membrane, enabling their easier release into the cytoplasm [195]. However, most of these techniques have only been tested in vitro, since drugs in clinical phases are mainly delivered with conventional methods (Table 9).

Several trials have investigated drug delivery methods. A novel example that has completed clinical Phase II is Atu-027 **(NCT00938574**, **NCT01808638)**. It is used for treating solid tumors and pancreatic cancer in combination with gemcitabine. This drug is delivered via liposomes, to silence the mRNA expression of protein kinase N3 (PKN3), a downstream effector of the PI3 pathway in the vascular endothelium. It is composed of four components: AtuRNA, which is a 23-mer blunt-ended chemically stabilized siRNA, a cationic lipid (AtuFECT01) used to target endothelial cells [196,197], a neutral helper lipid, and a pegylated lipid that offers stability [198]. This drug has a major difference in comparison to other drugs as it is diluted in 5% xylitol, whereas other liposome-based drugs require corticosteroids and H2 blockers as premedication to avoid serious immune stimulation, allergy, or other adverse effects. The Atu-027 liposomal siRNA complex delivers siRNAs into the cytoplasm of vascular endothelial cells in vivo for sequence-specific cleavage of the *PKN3* mRNA. When separately delivered, these compounds were shown to possibly activate the innate immune system; hence, it was necessary to investigate whether it would be activated when the whole complex is delivered. Importantly after measuring serum cytokines, no allergic reaction was noticed, thus suggesting that administration of immunosuppressive premedication is unnecessary [199].

### 6.2. Extracellular Vesicles (Exosomes)

Another drug delivery system refers to extracellular vesicles. These are naturally derived membrane structures forming a lipid bilayer [200]. They hold significant potential due to their low immunogenic properties and their ability to carry their cargo (nucleic acids, proteins, or drugs) to target cells with high specificity. This level of specialization can be achieved through modification of their surface with targeting peptides or ligands that specifically target cancer cells (Table 10) [201]. Another mechanism that provides a great advantage compared to other methods involves interaction with recipient cells; this can be achieved by incorporating receptors on EV surface, or scaffolding proteins to induce signaling such as tetraspanin proteins and adhesion molecules [201,202]. Moreover, engineering of extracellular vesicles (EVs) to display specific proteins, such as tumor-specific antigen peptides, can be used as tools to modulate immune responses [203]. Lastly, in the acidic environment of the tumor, incorporating high lipid raft composition influences EV cellular uptake [203,204]. EVs share common characteristics with their parental cells, so it is important to select the appropriate composition when designing a therapeutic strategy so as to avoid extra modifications without compromising passage through biological barriers [201,202].

EVs fall into three categories according to their size, biogenesis, and composition: exosomes, apoptotic bodies, and microvesicles [201]. They may induce resistance to chemotherapy, radiotherapy, and targeted therapy by carrying specific cargos that stimulate drug efflux through activation or suppression of signaling pathways associated with metabolism, autophagy, cancer stemness, and epithelial–mesenchymal transition (EMT) [202]. Interestingly, many ncRNAs were found to trigger resistance to a variety of drugs; thus, their silencing might restore sensitivity. Importantly, EVs can effectively encapsulate ncRNAs, which can be used to target drug-resistant genes in cancer. Drug resistance can also be reversed by inhibiting the biogenesis of extracellular vesicles and their subsequent release, or by blocking their internalization by target cells [205].

Many clinical investigations have assessed the efficacy of these platforms, one of which is iExosomes that carry siRNA (AF647 siRNA) or shRNA targeting of the *KRAS^G12D^* mRNA. Silencing of this mRNA, which is involved in proliferation pathways, resulted in impaired proliferation and enhanced apoptosis in pancreatic cancer cells, while leaving the wild-type KRAS (BxCP-3) unaffected, indicating that it controls tumor size growth. This exosome also contained CD47 protein, responsible for the “don’t eat me” signal to cells, thus avoiding phagocytosis. It was observed that micropinocytosis was increased in cancerous pancreatic cells in contrast to normal cells [206]. In the same study, iLiposomes were also used as a drug delivery method, but researchers showed that the presence of plasma membrane-anchored proteins and membrane-like phospholipids renders iExosomes as a better vector due to their increased half-life during circulation.

### 6.3. Non-Lipid Organic-Based Nanovectors

#### 6.3.1. Dendrimers

Dendrimers are a new class of biomaterials with great potential for drug delivery. They consist of three main parts: core, internal cavity, and surface [207]. They are classified according to their shape, their inner cavities, the surface groups that they carry, and the way that they are generated. The main types are polyamidoamine dendrimers (PAMAM), polypropyleneimine (PPI), poly-l-lysine (PLL), phosphorus-based, silicon-based, metallodendrimers, and triazine [208].

Their unique architecture, as highly branched, spherical, and tiny polymeric structures (1–100 nm), enables them to form organized structures that are soluble in water, with enhanced stability and great pharmacokinetics by decreasing renal clearance and immunogenicity [209,210]. Through their vast, adjustable surface, dendrimers can carry anticancer drugs via two main strategies: encapsulation within their internal hydrophobic voids or covalent conjugation to a wide range of therapeutic substances, such as nucleic acids, mAbs, folic acids, peptides, and chemotherapeutic drugs [208,210]. When dendrimers are conjugated, off-target effects are reduced, enabling controlled drug release; their biocompatibility, cellular uptake, and circulation time are enhanced, whereas their immunogenicity decreases [210,211]. In this way, systemic toxicity is reduced, and therapeutic outcomes are improved.

Dendrimer modifications that improve their physicochemical characteristics refer to PEGylation (covalently attaching polyethylene glycol to dendrimers), through which dendrimer solubility and biocompatibility are enhanced [211]. Due to their small size, PEGylated dendrimers can target cancer cells passively as the tumor tissues have a leaky vasculature system and poor lymphatic drainage [207]. Targeting can also occur actively through the conjugated ligands of the dendrimer that bind to the overexpressed receptors of the cancer cells [209]. Moreover, the cargo of the dendrimers can also be released as a response to stimuli, as the pH in the tumor microenvironment is low, and the oxidative stress is relatively high; in this way, healthy cells remain unharmed, and toxicity is reduced [209,211].

Another delivery approach called proton-sponge is also utilized, according to which polymers and peptides containing amino groups can be protonated in the acidic environment of endosomes, hence gaining a great buffering capacity. The acidification occurs due to the activity of an ATPase enzyme that transports protons from the cytosol to the surface of the vehicle. This leads to an influx of chloride counter ions with a subsequent increase in osmotic pressure inside the endosome. In response to this, the endosome starts swelling and inevitably its membrane is ruptured, releasing nanoparticles into the cytosol [195]. Another benefit of dendrimers associated with their small size is passage through various barriers, thus enhancing delivery to the complex microenvironment of tumors; for that reason, they are mainly applied in brain and gastrointestinal types of cancer [208,210]. One way to surpass the blood–brain barrier is usage of transferrin-conjugated PEGylated dendrimers, as they target the brain capillary endothelial and cancer cells that internally express transferrin receptors, and thus penetration through the blood–brain barrier.

Dendrimers are used in clinical cancer treatment, but they mostly deliver synthetic anticancer drugs; very few that contain ncRNAs have passed the clinical Phase I. AZD0466 **(NCT05205161)**, a drug–dendrimer conjugate of the BCL2/BCL-XL inhibitor based on AZD4320 (a potent Bcl-2/Bcl-xL dual inhibitor) [211,212]. In leukemia, the anti-apoptotic proteins B-cell lymphoma 2 (BCL2) and B-cell lymphoma extra-large (BCL-XL), which normally act as regulators of apoptosis, are overexpressed; in this way, they allow leukemia cells to evade apoptosis, leading to tumorigenesis and chemoresistance [213]. The pharmacokinetics and safety of this drug were also tested in patients with advanced hematologic or solid tumors **(NCT04214093)** and patients with advanced hematological malignancies **(NCT04865419)** [212]. Overall, dendrimers seem to carry great promise in future applications, considering their unique characteristics and great potential of targeting a variety of organs.

Another drug that has reached clinical Phase I/II is mRNA encoding Wilms’ Tumor 1 protein (WT1) to treat acute myeloid leukemia **(NCT01686334)**. It is a novel example of utilizing a tumor antigen to activate a specific T-cell response. Indeed, in response to vaccination, elevated levels of WT1-specific CD8+ T-cells were found, while natural killer cells were also activated. This observation indicated that vaccination with *WT1* mRNA-loaded dendritic cells might aid in preventing full relapse in acute myeloid leukemia patients [214].

#### 6.3.2. Polymeric Nanoparticles

Polymeric nanoparticles, also defined as “colloidal macromolecules” consist of either synthetic or natural polymers. They have many advantages over conventional LNPs, as they can incorporate ligands, they are cheaper to produce, and their physicochemical properties are adjustable [215]. Most commonly, polymeric nanoparticles include modified natural polymers such as chitosan, cellulose, dextran, gelatin, collagen, and synthesized polymers like poly lactic acid (PLA), poly ε-caprolactone, or their PEGylated forms [215]. Selected examples of this delivery platform are shown in Table 11.

One drawback of polymeric nanoparticles is the lack of a cationic moiety, which prevents direct encapsulation of RNA [215]. This is why cationic motifs like polyethyleneimine are incorporated in the structure of polymeric nanoparticles, while addition of organelle-targeting peptides increases their subcellular targeting [215]. With regard to their size, it ranges from 1 nm to 1000 nm [216]. Drug compounds are either encapsulated in their cavity or attached to their surface, creating a nanosphere or a nanocapsule to reach the targeting site and accomplish controlled release [217]. This can happen by incorporating disulfide links that can be reduced by intracellular GSH (2–10 mM) while they are stable in the extracellular environment (~2–20 μM). In this way, premature drug release is prevented [218]. In addition, another developed technique is incorporation of PEG via metalloprotease-sensitive peptides. In this way, when entering an MMP-rich environment, such as the tumor, nanoparticles lose their PEG coating, and their cationic core is exposed. After that, ncRNAs are encapsulated, and cellular internalization takes place [218].

The ideal diameter of drug delivery carriers in vascular tumors is 100 nm to allow passage through fenestrations in the endothelium of a blood vessel, whereas for brain tumors, even smaller diameters (~70 nm) are required to surpass the blood–brain barrier [219]. Recently, albumin has been widely used as a soluble, stable, non-immunogenic, and biodegradable protein. Another advantage is that albumin has numerous binding sites in the body along with a long half-life (~19 days). When it interacts with epithelial cell surface receptors, it can be actively transported via transcytosis (both endocytosis and exocytosis). Thus, it can transport drugs to solid tumors without the need of using antibodies or other ligands/peptides for cellular entry [220,221]. Another great application is incorporating chitosan onto the surface of poly(lactide-co-glycolactide) (PLGA) nanoparticles, as it enhances their ability to bind antisense oligonucleotides and improves their cellular absorption [222].

CALAA-01, a Phase I clinical trial **(NCT00689065)**, was conducted to treat solid tumors using a nanoparticle delivery system. [223] Here the nanoparticle consists of (i) a linear cyclodextrin-based polymer, (ii) a human transferrin protein-targeting ligand that binds to the TF receptors of the cancer cells (known to be overexpressed in cancer cells) [224], (iii) a hydrophilic polymer, polyethylene glycol (PEG) that enhances the stability of the nanoparticle in the circulation, and (iv) the siRNA, which is designed to reduce the expression of RRM2 which is a common anticancer target [225].

### 6.4. Other Delivery Methods

Apart from the strategies described above, other methods for drug delivery have also been developed (Table 12). For example, viral vectors were the first delivery platform used for gene therapy, as they are appealing for DNA and RNA delivery in human cells. As a result, many Phase I/II clinical trials, such as **NCT03240861** and **NCT03250325**, were conducted, in which retroviral vectors served as the delivery method. Through them, silencing of the NY-ESO-1-specific T-cell receptor (TCR) with TBI-1301 siRNA was achieved in patients with solid tumors [226,227]. However, increased immunogenicity along with the limited genetic load of this method are some factors that should be taken into consideration when designing drug delivery vectors, and for that reason, nanoparticles are more preferable [187].

Another method used is referred to as locked nucleic acids (LNA). LNAs are a new generation of ASOs in which the ribose moiety of an LNA nucleotide is modified with an extra bridge connecting the 2′ oxygen and 4′ carbon nucleoside [228]. This slight modification endows oligonucleotides with a high affinity for mRNA bonding in contrast to conventional ASOs. Another advantage is resistance to nucleases, providing this delivery platform with great stability in plasma [229]. As for cellular uptake, naked LNAs primarily enter the cell via endocytosis. The mechanism of their release is yet unknown; nonetheless, once they escape from the endosome, they are released in the cytoplasm, where they can bind to their targets [228]. A Phase I clinical trial was conducted regarding *miR-221* **(NCT04811898)** as a possible treatment for advanced solid neoplasms. The drug contained a 13-mer LNA inhibitor against *miR-221* with a full phosphorothiate-modified backbone, and the study aimed to evaluate dose escalation of the drug. No adverse effects were noticed, and the safety of this study enables the use of LNA and *miR-221* for future developments [157].

Aptamers are small single-stranded DNA or RNA oligonucleotides that can be designed to function as highly specific and sensitive ligands. They have low immunogenicity, thus leading to better organ distribution and circulating stability in the organism [230]. Among their unique characteristic is their three-dimensional folding, which is a result of intramolecular interactions. Their structure contains ligand-binding characteristics, while their high level of specificity is the outcome of an in vitro chemical process known as “systemic evolution of ligands by exponential enrichment” (SELEX). In this process, a library containing random oligonucleotides is designed to specifically target the sequence of interest [231]. Furthermore, due to their small (~20 kDa) molecular weight, aptamers can easily penetrate tumor cells. Moreover, they are easily produced, thus making a good candidate for drug delivery. Their limitation lies in the fact that they are very easily degradated inside the organism [232].

An ongoing clinical trial Phase I/II regarding NOX-A12 **(NCT04121455)** and **(NCT04901741)**, which is delivered via aptamers to treat chronic lymphocytic leukemia (CLL), took place. NOX-A12 is an RNA oligonucleotide that binds to CXCL12. Its binding capacity is based on its L-configuration, mirroring CXCL12 (D-configuration according to Spiegelmer). It has an antagonistic role with glycosaminoglycans to bind CXCL12. Normally, stromal cells secrete CXCL12, activating CKCR4 and attracting CLL cells to protect them from cytotoxic drugs. Due to configuration that mirrors a natural ligand, NOX-A12 does not induce immunostimulation and is not susceptible to endonucleases. Binding of NOX-A12 to CXCL12 inhibits CLL migration, activating mobilization of hematopoietic stem cells, resulting into a strong therapeutic effect [233].

Finally, gold nanoparticles (AUNPs) are the most widely used metallic nanoparticles due to their biocompatibility, stability, and ease of preparation [234]. Their size ranges from 1 to 100 nm, while their unique physical and chemical characteristics, obtained from nano-ionization, make them suitable for cancer applications [235]. A great benefit is that they can be engineered to selectively target cancer cells. Their applicability is based on their ability to shine light on cancer cells for subsequent detection and destruction. To achieve this, it is necessary to deliver the right wavelength of light to the right position [236]. A Phase I clinical trial aiming to treat glioblastoma using NU-0129 AUNPs as a drug delivery nanoparticle was approved **(NCT03020017)**. The molecular target of NU-0129 was Bcl2L12 cells, which are overexpressed in glioblastoma and show great resistance to apoptosis. Importantly due to their small size, AUNPs can cross the blood–brain barrier, yet their clinical application is hindered by several challenges, as the reproducible scale-up is limited and their long-term biological fate is unknown [237]. Therefore, additional research is required to assess the clinical safety of this therapeutic scheme [238].

## 7. Limitations and Challenges

**Figure 5:**
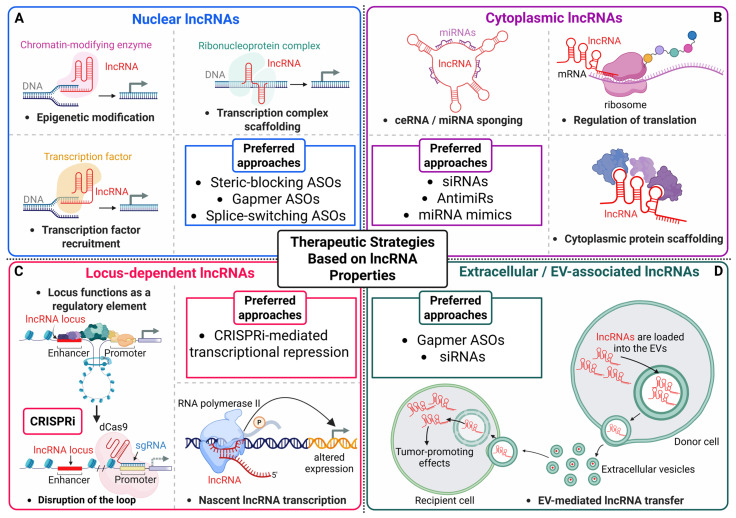
Therapeutic strategies according to the biological properties of lncRNAs. (**A**) Nuclear lncRNAs primarily regulate epigenetic modifications, transcription complex scaffolding and transcription factor recruitment, making steric-blocking ASOs, gapmer ASOs and splice-switching ASOs the preferred therapeutic approaches. (**B**) Cytoplasmic lncRNAs function through mechanisms such as miRNA sponging, regulation of translation and cytoplasmic protein scaffolding, and are therefore generally amenable to siRNAs, antimiRs and miRNA mimics. (**C**) Locus-dependent lncRNAs exert their functions through their genomic loci or nascent transcription rather than through the mature transcript. Consequently, CRISPRi-mediated transcriptional repression is the preferred therapeutic strategy, as it disrupts locus-dependent regulatory activity. (**D**) Extracellular/EV-associated lncRNAs mediate intercellular communication through extracellular vesicle (EV)-mediated transfer. This process may be reduced by intracellular depletion of oncogenic lncRNAs using gapmer ASOs or siRNAs, thereby limiting lncRNA loading into EVs and subsequent transfer to recipient cells. Abbreviations: ceRNA = competing endogenous RNA, CRISPRi = CRISPR interference, EV = extracellular vesicles. Created in BioRender. (2026) https://BioRender.com/td5o982.

Despite the growing evidence supporting lncRNAs as therapeutic candidates in cancer, several considerable limitations remain. First, not all lncRNAs are equally suitable for therapeutic targeting. Unlike protein-coding transcripts and conventional regulatory RNAs, lncRNAs represent a heterogeneous class with multimodal properties that largely determine the optimal intervention strategy (Figure 5) [239]. Moreover, lncRNAs frequently regulate broad gene expression networks rather than single oncogenic targets [240,241,242]. To enhance therapeutic response with minimum toxicity, future lncRNA-based therapies need to take into consideration properties, such as subcellular localization (e.g., cytoplasm or nucleus) [11], mechanism of function (e.g., transcriptional scaffolding for proteins or post-transcriptional RNA::RNA interactions), post-transcriptional stability of the lncRNA (e.g., short vs. long transcript half-life), tumor specificity (e.g., lineage organ specificity or wide-spread presence in cancer tissues) before applying any of the available RNA-based therapeutic strategies (Figure 5) [13].

For example, nuclear-enriched lncRNAs, including chromatin-associated transcripts that regulate transcriptional complexes, epigenetic modifiers, or transcription factor recruitment, are unlikely to be efficiently targeted by conventional siRNA approaches because of limited cytoplasmic access of the RISC. Instead, steric-blocking ASOs, particularly RNase H–recruiting gapmer ASOs [89,243], represent a more suitable approach because they can enter the nucleus and induce degradation of target lncRNAs through an RNase H-dependent mechanism. Similarly, splice-switching ASOs [244] may be applicable to nuclear lncRNAs that regulate alternative splicing or transcript processing by altering RNA–protein interactions or splice-site accessibility.

In contrast, cytoplasmic lncRNAs, including transcripts functioning as ceRNAs, microRNA sponges, translational regulators, or protein scaffolds, may be more amenable to siRNA-mediated degradation, although therapeutic efficacy will depend on transcript abundance, turnover rate, and the degree to which the lncRNA function depends on sequence rather than structural interactions. For cytoplasmic lncRNAs acting primarily as miRNA decoys, indirect modulation through antimiRs or miRNA mimics represents a potential targeting strategy; however, these approaches target the broader competing RNA network rather than the lncRNA itself and therefore require careful evaluation of pathway-level consequences [109,245].

While classical lncRNAs can often be inhibited through transcript-directed strategies, certain lncRNAs exert their biological functions through their genomic loci, nascent transcription or local regulatory elements rather than through the mature RNA transcript itself [241]. In these cases, depletion of the lncRNA transcript may not fully reproduce the consequences of targeting the entire regulatory locus. Such locus-dependent lncRNAs, require endogenous transcriptional repression approaches analogous to CRISPR interference (CRISPRi), without inducing DNA cleavage. Effective delivery is another major determinant of therapeutic feasibility: lipid nanoparticles, EVs, polymeric nanoparticles, and other delivery systems may provide tissue-selective access, but their applicability will likely vary according to the biological compartment in which the target lncRNA operates [245].

Extracellular and exosome-associated lncRNAs constitute a distinct therapeutic category because their pathogenic effects frequently arise from intercellular transfer rather than solely from intracellular activity [246]. Tumor-derived EVs transport lncRNAs to neighboring cancer cells, stromal fibroblasts, endothelial cells, and immune cells, where they can remodel the tumor microenvironment, promote epithelial-to-mesenchymal transition, stimulate angiogenesis, facilitate immune evasion, and disseminate drug resistance. Consequently, future therapeutic strategies should aim not only to reduce lncRNA expression within donor tumor cells but also to disrupt their extracellular trafficking and uptake. Intracellular depletion using gapmer ASOs or siRNAs may decrease the loading of oncogenic lncRNAs into EVs, whereas approaches targeting EV biogenesis, cargo sorting, secretion, or cellular uptake may further limit intercellular dissemination.

It should be noted that emerging strategies, including engineered EVs for targeted nucleic acid delivery, antibody- or ligand-mediated interception of tumor-derived vesicles, and circulating oligonucleotides capable of neutralizing extracellular RNAs, remain largely preclinical [247], yet highlight the potential to selectively interfere with lncRNA-mediated cell-to-cell communication. Another concern refers to safety and toxic side-effects, since systemic inhibition of EV trafficking could disrupt physiological extracellular vesicle signaling involved in tissue homeostasis, immune regulation, and regeneration. Therefore, strategies directed against specific oncogenic lncRNA cargo or tumor-selective EV populations are likely to offer a more favorable therapeutic index than global suppression of extracellular vesicle production.

The mechanism of tumor-dependent lncRNA operation along with their stability also influence therapeutic selection: as mentioned, lncRNAs act as regulatory network hubs, raising concerns that their inhibition may produce unintended effects on normal cellular pathways [242,248]. Stable lncRNA transcripts that function as oncogenic scaffolds or transcriptional regulators may require sustained depletion using gapmer ASOs or transcriptional repression strategies, whereas lncRNAs acting as competitors of endogenous RNAs or as microRNA sponges may require modulation of the broader regulatory network through antimiRs, miRNA mimics, or conventional pathway inhibition. Importantly, anticipated toxicities are expected to differ between strategies and therefore among lncRNA targets; ASOs targeting nuclear lncRNAs, may induce immune activation, thrombocytopenia, or unintended RNA binding effects, while siRNAs targeting cytoplasmic lncRNAs may produce sequence-dependent off-target gene silencing and innate immune responses. miRNA-based approaches may perturb multiple downstream transcripts due to the broad regulatory nature of miRNA networks, while CRISPRi-based approaches require assessment of genomic targeting specificity and long-term effect evaluation before even entering clinical trials.

An additional consideration for lncRNA-directed therapeutics is their tumor specificity, which may strongly influence both therapeutic index and safety profile [13]. Unlike typical protein-coding oncogenic drivers that are broadly conserved across malignancies, lncRNAs frequently display context-dependent expression patterns and functions [11,249]. Pan-cancer lncRNAs that are recurrently activated across multiple tumor types may represent attractive therapeutic candidates because of their broad applicability; however, their expression in normal tissues or their involvement in fundamental cellular processes may increase the risk of systemic toxicity. For these transcripts, approaches such as tumor-selective delivery vehicles, ligand-directed nanoparticles, or transient RNA degradation strategies may be required to achieve an acceptable therapeutic window. Conversely, lineage-restricted lncRNAs that are preferentially expressed in specific malignancies, such as gastric-, esophageal-, hepatic-, or hematopoietic-associated lncRNAs, may provide greater opportunities for precision targeting because inhibition may spare normal tissues. However, their restricted expression introduces challenges related to patient stratification, biomarker development, and intertumoral heterogeneity, as dependence on a particular lncRNA may be limited to specific molecular subtypes or disease states.

Several limitations also arise from the delivery systems of RNA therapeutics [245]. RNA-based strategies may produce incomplete target silencing, which can weaken therapeutic efficacy [59]. One way to address this is to increase RNA cargo quantities, which in turn poses new challenges through induction of immune activation or systemic toxicity, highlighting the need for careful chemical optimization and safety assessment [250]. Specificity of delivery remains another major barrier, as therapeutic RNA molecules must be efficiently loaded into carriers, survive circulation, avoid renal clearance, reach specific organs or tumor cell populations, enter target cells, and effectively release their cargo intracellularly [250]. However, tumor- and organ-specific delivery remain difficult, and many delivery platforms still face challenges related to pharmacokinetics, short circulation half-life, cytotoxicity, immunogenicity, endosomal escape, and limited ability to cross biological constrains such as the blood–brain barrier [165,251]. In addition, many nanocarriers do not naturally possess cationic moieties, limiting direct binding of negatively charged RNA cargo [215]. Furthermore, although several delivery technologies have shown promising performance in experimental models, many remain primarily validated in vitro, whereas clinical-stage RNA therapeutics still frequently rely on more conventional delivery approaches. Their clinical translation is further complicated by challenges in reproducible large-scale production and by the potential for adverse effects, particularly when repeated or long-term administration is required [252].

Consequently, unlike conventional RNA therapeutics where target abundance is often the primary determinant of efficacy, successful lncRNA targeting and thus future development of lncRNA therapeutics will require integration of lncRNA localization, mechanism of action, disease-specific expression patterns, transcript- versus locus-dependent function, functional dissection of downstream pathways affected by lncRNA inhibition and delivery constraints rather than direct extrapolation from existing mRNA, miRNA, or protein-targeting RNA medicines. Addressing these biological, pharmacological, and delivery-related barriers will be essential for translating lncRNA-targeted designs into clinically effective anticancer strategies.

## 8. Conclusions and Future Perspectives

This review focuses on investigating the molecular mechanisms and the potential of ncRNA-based cancer therapeutics that reveal an unexplored potential in overcoming chemotherapy resistance. The problem with conventional cancer therapies is that, initially, patients respond to the treatment, but eventually develop resistance to drugs as a result of alterations in the tumor microenvironment as well as due to genetic changes, resulting in drug efflux. To address this issue, research has been initiated into effective delivery strategies for ncRNA-based cancer therapeutics. Backbone modifications in ncRNAs are an interesting engineering technique that will facilitate RNA therapeutics in multiple ways, including but not limited to prolongation of their half-life, increase in cellular uptake and stability, reduction in immunogenicity and improvement of target specificity. More specifically, lncRNAs have the potential to interfere with several molecular pathways and, in this way, retain sensitivity to chemoresistance drugs, while improving immunotherapy and radiotherapy results. Despite the fact that some studies correlate lncRNAs with therapeutic outcomes, additional research is required to verify the direct relationship between lncRNAs and therapeutic success.

Advances in drug delivery technologies are transforming ncRNAs from experimental molecules into viable therapeutic agents. Established methodologies such as SORT and SELEX, along with the combination of hybrid nanoparticles including both lipid and polymeric materials, will most likely overcome limitations of drug delivery platforms. LNPs can overcome cellular uptake-related barriers, whereas the application of polymeric nanoparticles will enhance the drug’s pharmacokinetic profile [253]. Modification of ncRNAs, usage of ligands on the surface of nanoparticles, and integration of local stimuli to release the drug in the cell in response to a specific cellular environment hold great potential for future targeted approaches. The exploitation of biomimetic nanoparticles, such as EVs, is a promising strategy to overcome immunogenicity-related obstacles. Ultimately, the convergence of ncRNA biology, nanoparticles, and personalized treatment has the potential to redefine the landscape of future anticancer therapies.

## Figures and Tables

**Table 1:** Selected examples of lncRNAs involved in radioresistance.

lncRNA	Cancer Type	Mechanism	Effect	Ref.
*SP100-AS1* (OE)	CRC	Sponges *miR-622* stabilizing *ATG3* mRNA	Increased autophagic flux	[24]
*linc00312 *(UE)	NPC	Binds to DNA-PKcs inhibiting Ku complex recruitment	Impaired DNA damage sensing and repair	[25]
*HOTAIRM1 *(OE)	NPC	Enhances FTO protein stability via acetylation, shifting CD44 splicing toward CD44V isoform	Inhibition of ferroptosis	[26]
*WARS2-IT1 *(OE)	CRC	Enhances HIF-1α protein stability by disrupting its interaction with PHD2	Activation of glycolytic pathways	[27]

lncRNA = long non-coding RNA, OE = overexpressed in resistant tumors, UE = underexpressed in resistant tumors, mRNA = messenger RNA, CRC = colorectal cancer, NPC = nasopharyngeal carcinoma, ATG3 = Autophagy-related 3, DNA-PKcs = catalytic subunit of DNA-dependent protein kinase, HIF-1α = hypoxia-inducible factor 1 alpha, PHD2 = prolyl hydroxylase domain protein 2.

**Table 2:** Selected examples of lncRNAs involved in chemoresistance.

lncRNA	Cancer Type	Therapy	Mechanism	Effect	Ref.
*NEF* (UE)	CRC	Oxaliplatin	Prevents DNMT-mediated *DOK1* gene silencing	Activation of MEK/ERK pathway	[30]
*GDIL *(OE)	CRC	Oxaliplatin	Promotes XRN2-mediated *CHAC1* mRNA degradation	GSH levels preservation	[31]
*GAS6-AS1 *(OE)	CRC	5-FU	Promotes PCBP1-mediated stabilization of *MCM3* mRNA	Increased cell growth	[32]
*FUAT1 *(OE)	GC	5-FU	Sponges *miR-140-5p* upregulating *TNS4* mRNA expression	Inhibition of apoptosis	[33]
*HMG *(OE)	CRC	Oxaliplatin, 5-FU	Promotes MDM2-mediated p53 protein degradation	Inhibition of ferroptosis	[34]
*CACClnc *(OE)	CRC	Oxaliplatin, 5-FU	Modulates *RAD51* mRNA alternative splicing	Enhanced DNA repair	[35]
*STMN1P2 *(OE)	BRCA	Doxorubicin	Interacts with hnRNPU protein, inactivating caspase-1-dependent pathways	Inhibition of pyroptosis	[36]
*LUCAT1 *(OE)	BC	Gemcitabine	Enhances m6A-mediated *HMGA1* mRNA stability	Maintenance of stemness	[37]
*DDIT4-AS1 *(OE)	TNBC	Paclitaxel	Enhances *DDIT4* mRNA stability via promoting its interaction with AUF1	Promotion of autophagy	[38]
*NEAT1 *(OE)	BRCA	Paclitaxel	Sponges *miR-133b* upregulating *CXCL12* expression	Increased cell proliferation, invasion and metastasis	[39]

GC = gastric cancer, BRCA = breast cancer, BC = bladder cancer, TNBC = triple negative breast cancer, 5-FU = 5-fluorouracil, DNMT = DNA methyltransferase, DOK1 = downstream of tyrosine kinase 1, GSH = glutathione, MCM3 = Minichromosome Maintenance Complex 3, TNS4 = tension focal adhesion 4, m6A = N^6^-methyladenosine.

**Table 3:** Selected examples of lncRNAs involved in immunotherapy resistance.

lncRNA	Cancer Type	Therapy	Mechanism	Effect	Ref.
*LINC02544* (OE)	TNBC	Pembrolizumab (ICI)	Sponges *miR-497-5p* upregulating CAPRIN1 expression	Increased proliferation and migration	[49]
*EPIC1 *(OE)	TNBC	Pembrolizumab (ICI)	Represses retroelements via EZH2 recruitment, reducing dsRNA and IFN signaling	Reduced immune activation and T-cell infiltration	[50]
*LINK-A *(OE)	TNBC	Pembrolizumab (ICI)	Promotes TRIM71-mediated PLC degradation	Reduced MHC-I antigen presentation and CD8+ T-cell recognition	[51]

ICI = immune checkpoint inhibitor, dsRNA = double-stranded RNA, IFN = type I interferon, PLC = peptide-loading complex, MHC-I = major histocompatibility complex class I.

**Table 4:** Selected examples of lncRNAs involved in targeted therapy resistance.

lncRNA	Cancer Type	Therapy	Mechanism	Effect	Ref.
*Linc00969* (OE)	BRCA	Trastuzumab (mAb)	Promotes the interaction between *HER2* mRNA and HuR, increasing *HER2* mRNA stability	Increased tumor growth and autophagy	[53]
*MALAT1 *(OE)	BRCA	Trastuzumab (mAb)	Enhances m6A-mediated *N-HER2* stability	Increased proliferation and migration	[54]
*UCA1 *(OE)	CRC	Cetuximab (mAb)	Sponges *miR-495*, upregulating HGF and c-MET stability leading to activation of PI3K/AKT and MAPK signaling	Increased proliferation and cell survival	[55]
*HNF4A-AS1 *(UE)	HCC	Sorafenib (SMKI)	Reduces m6A-mediated *DECR1* degradation	High PUFA levels, inhibition of ferroptosis	[56]
*LINC01056 *(UE)	HCC	Sorafenib (SMKI)	Interacts with PPARα promoting its nuclear translocation	Metabolic shift to FAO, tumor survival	[57]
*EILA *(OE)	BRCA	Palbociclib (SMKI)	Enhances cyclin E1 protein stability by preventing its interaction with FBXW7	Cell cycle progression to S-phase	[58]

mAb = monoclonal antibodies, SMKI = small molecule kinase inhibitors, HCC = hepatocellular carcinoma, HER2 = human epidermal growth factor receptor 2, HuR = Hu antigen R, N-HER2 = nuclear human epidermal growth factor receptor 2, HGF = hepatocyte growth factor, c-MET = c-mesenchymal–epithelial transition, PPARα = peroxisome proliferator-activated receptor alpha, FAO = fatty acid oxidation, PUFA = polyunsaturated fatty acid.

**Table 5:** Regulatory milestones of approved RNA therapeutics.

Name	Type	Target	Administration	Target Organ	Disease	Phase	Ref.
Fomivirsen	ASO	*CMV* mRNA	Intravitreal	Eye	Cytomegalovirus retinitis	FDA (1998)- and EMA (1999)-approved, currently withdrawn	[63]
Mipomersen	ASO	Apolipoprotein *B-100* mRNA	Subcutaneous	Liver	Homozygous familial hypercholesterolemia	FDA (2013)- and EMA (2012)-approved, currently withdrawn	[64]
Nusinersen	ASO	*SMN2* pre-mRNA	Intrathecal	CNS	Spinal muscular atrophy	FDA (2016)- and EMA (2017)-approved, currently marketed	[65]
Eteplirsen	ASO	Exon 51 of dystrophin pre-mRNA	Intravenous	Muscle	Duchenne muscular dystrophy	FDA (2016)-approved, currently marketed	[66]
Inotersen	ASO	*TTR* mRNA	Subcutaneous	Liver	Hereditary transthyretin amyloidosis	FDA- and EMA (2018)-approved, currently marketed	[67]
Golodirsen	ASO	Exon 53 of *DMD*	Intravenous	Muscle	Duchenne muscular dystrophy	FDA (2019)-approved, currently marketed	[68]
Viltolarsen	ASO	Exon 53 of dystrophin pre-mRNA	Intravenous	Muscle	Duchenne muscular dystrophy	FDA (2020)-approved, currently marketed	[69]
Volanesorsen	ASO	*Apolipoprotein CIII* mRNA	Subcutaneous	Liver	Familial chylomicronaemia syndrome	EMA (2019)-approved, currently marketed	[70]
Milasen	ASO	*SMN2* pre-mRNA	Intrathecal	CNS	Neuronal ceroid lipofuscinosis 7	FDA (2019)-approved, currently marketed	[71]
Casimersen	ASO	Exon 45 of dystrophin pre-mRNA	Intravenous	Muscle	Duchenne muscular dystrophy	FDA (2021)-approved, currently marketed	[72]
Tofersen	ASO	*SOD1* mRNA	Intrathecal	CNS	Amyotrophic lateral sclerosis	FDA (2023)- and EMA (2024)-approved, currently marketed	[73]
Eplontersen	ASO	*TTR* mRNA	Subcutaneous	Liver	Polyneuropathy of hereditary transthyretin-mediated amyloidosis	FDA (2023)- and EMA (2024)-approved, currently marketed	[74]
Olezarsen	ASO	*Apolipoprotein CIII* mRNA	Subcutaneous	Liver	Familial chylomicronaemia syndrome	FDA (2024)- and EMA (2025)-approved, currently marketed	[75]
Imetelstat	ASO	RNA template of telomerase enzyme	Intravenous	Blood	Myelodysplastic syndromes	FDA (2024)-approved, currently marketed	[76]
Donidalorsen	ASO	*PKK* mRNA	Subcutaneous	Liver	Hereditary angioedema	FDA (2025)-approved, currently marketed	[77]
Patisiran	siRNA	*TTR* mRNA	Intravenous	Liver	Hereditary transthyretin amyloidosis	FDA (2019)- and EMA (2018)-approved, currently marketed	[78]
Givosiran	siRNA	*ALS1* mRNA	Subcutaneous	Liver	Acute hepatic porphyria	FDA (2019)- and EMA (2020)-approved, currently marketed	[79]
Inclisiran	siRNA	*PCSK9* mRNA	Subcutaneous	Liver	Atherosclerotic cardiovascular disease, elevated cholesterol, homozygous/heterozygous familial hypercholesterolaemia	EMA (2020)-approved, currently marketed	[80]
Lumasiran	siRNA	*HAO1* mRNA	Subcutaneous	Liver	Primary hyperoxaluria type 1	FDA (2020)- and EMA (2020)-approved, currently marketed	[81]
Vutrisiran	siRNA	*TTR* mRNA	Subcutaneous	Liver	Polyneuropathy of hereditary transthyretin-mediated amyloidosis or cardiomyopathy of wild-type or hereditary transthyretin-mediated amyloidosis	FDA (2022)- and EMA (2022)-approved, currently marketed	[82]
Nedosiran	siRNA	Hepatic *LDH* mRNA	Subcutaneous	Liver	Primary hyperoxaluria type 1	FDA (2023)-approved, currently marketed	[83]

ASO = antisense oligonucleotide, FDA = Food and Drug Administration, EMA = European Medicines Agency, CNS = central nervous system, TTR = transthyretin, siRNA = small interfering RNA. **Regulatory status definitions:** “Approved” indicates authorization by a regulatory agency for clinical use. “Withdrawn” refers to products that previously received regulatory approval but were subsequently removed from commercial availability or authorization. “Currently marketed” refers to commercially available products.

**Table 6:** Investigational RNA therapeutics evaluated in oncology clinical trials.

Name	Type	Target	Administration	Cancer Target	Phase	Clinical Trials ID
BP1001	ASO	*Grb2* mRNA	Intravenous	Acute myeloid leukemia, chronic myeloid leukemia	Phase II	NCT01159028, NCT04196257, NCT02781883
AZD9150	ASO	*STAT3* mRNA	Intravenous	Metastatic NSCLC, resectable early-stage NSCLC, pancreatic cancer, mismatch repair-deficient CRC	Phase II	NCT03819465, NCT03794544, NCT02983578
OGX-427	ASO	*Hsp27* mRNA	Intravenous	Squamous cell lung cancer, non-squamous NSCLC, urological neoplasms, metastatic bladder cancer, urinary tract neoplasms, castration-resistant prostate cancer	Phase II	NCT01120470, NCT01454089, NCT01829113, NCT02423590, NCT01780545
AZD5312	ASO	*AR* mRNA	Intravenous	Metastatic castration-resistant prostate cancer	Phase Ib/II	NCT02144051, NCT03300505
AZD4785	ASO	*KRAS ^G12D^* mRNA	Intravenous	Advanced solid tumors, NSCLC, metastatic pancreatic cancer	Phase I	NCT03101839, NCT03608631
AP12009	ASO	*TGF-β2* mRNA	Intravenous	Colorectal neoplasms, melanoma, pancreatic neoplasms, glioblastoma, advanced PD-L1-positive NSCLC	Phase IIb	NCT00844064, NCT00431561, NCT06579196
ASO-2039	Exosome-delivered ASO	*STAT6* mRNA	Intravenous	Advanced HCC and liver metastases from either primary GC or CRC	Phase I	NCT05375604
siG12D LODER	siRNA	*KRAS^G12D^* mRNA	Intratumoral	Advanced pancreatic cancer	Phase II	NCT01188785, NCT01676259
ATU027	siRNA	*PKN3* mRNA	Intravenous	Advanced Solid Cancers, advanced or metastatic pancreatic adenocarcinoma	Phase I/IIa	NCT00938574, NCT01808638
TKM-080301	siRNA	*PLK1* mRNA	Intravenous	Neuroendocrine tumors, adrenocortical carcinomas, primary and secondary liver cancer	Phase I/II	NCT01437007, NCT01262235, NCT02191878
EPHARNA	siRNA	*EphA2* mRNA	Intravenous	Advanced malignant solid neoplasm	Phase I	NCT01591356
LNA-i-miR-221	AntimiR	*miR-221*	Intravenous	Advanced solid tumors, multiple myeloma, refractory hepatocarcinoma	Phase I	NCT04811898
MRG-106	AntimiR	*miR-155*	Intravenous	Cutaneous T-cell lymphoma (Mycosis Fungoides Subtype), DLBCL	Phase I/II	NCT02580552, NCT03713320
MRX34	miRNA mimic	*miR-34a*	Intravenous	Liver cancer, NSCLC, renal cell carcinoma, lymphoma, melanoma, advanced solid tumors	Phase I/Ib	NCT01829971, NCT02862145
TARGOmiR	miRNA mimic	*miR-16*	Intravenous	Malignant pleural mesothelioma, NSCLC	Phase I	NCT02369198
INT-1B3	miRNA mimic	*miR-193a*	Intravenous	Advanced solid tumors	Phase I/Ib	NCT04675996

NSCLC = non-small-cell lung cancer, Hsp27 = heat shock protein 27, PKN3 = protein kinase N3, miRNA = microRNA.

**Table 7:** Selected lncRNAsevaluated in preclinical cancer models as potential therapeutic candidates or biomarkers.

lncRNA	Therapy	Cancer Type	Treatment	Model	Ref.
*lncRNA16*	cisplatin	NSCLC	*miRNA1827* agomir (miRNA mimic)	Cell lines, CDX, PDX	[158]
*AC104041.1*	Wnt inhibitors	HNSCC	LNA-modified ASOs	Cell lines, PDX	[159]
*LOC644656*	5-FU	LIHC	ASOs	Cell lines, 3D culture, CDX	[160]
*MALAT1*	oxaliplatin	GC	siRNAs	Cell lines, CDX	[161]
*PURPL*	doxorubicin	HCC	ASOs	Cell lines	[162]
*HOTAIRM1*	radiotherapy	NPC	ASOs	Cell lines, CDX	[26]
*GDIL*	oxaliplatin	CRC	ASOs	Cell lines, CDX, PDX	[31]
*LINC02544*	pembrolizumab (ICI)	TNBC	siRNAs	Cell lines, CDX	[49]
*GAS6-AS1*	5-FU	CRC	ASOs	Cell lines, PDX	[32]
*lncRNA-HMG*	5-FU and oxaliplatin	CRC	ASOs	Cell lines, PDX	[34]
*EILA*	palbociclib (SMKI)	BRCA	ASOs	Cell lines, CDX	[58]

CDX = cell line-derived xenograft, PDX = patient-derived xenograft, HNSCC = head and neck squamous cell carcinoma, LNA = locked nucleic acid, LIHC = liver hepatocellular carcinoma.

**Table 8:** (**a**) Selected examples of RNA silencing therapeutics in oncology clinical trials. (**b**) Selected examples of mRNA immunotherapies in oncology clinical trials.

**(a)**						
**Name**	**Target**	**Administration**	**Delivery Method**	**Type of Cancer**	**Phase**	**Clinical Trials ID**
TKM-080301	PLK1	Intravenous	LNP-stable nucleic acid	Adrenocortical carcinoma & neuroendocrine tumor, HCC	Phase I/II	NCT01262235, NCT02191878
EPHARNA	EphA2	Intravenous	LNP-based siRNA	Advanced malignant solid neoplasm	Phase I	NCT01591356
ALN-VSP02	KSP and VEGF-A	Intravenous	LNP-ALN	Advanced solid tumor with liver involvement	Phase I	NCT00882180, NCT01158079
DCR-MYC	MYC	Intravenous	LNP-DCR	Solid tumor, multiple myeloma, or lymphoma	Phase I	NCT02110563
AtuPLEX (NBF-006)	PKN3, NBF-006	Intravenous	Lipoplex nanoparticle	NSCLC, pancreatic, or CRC	Phase I	NCT03819387
OGX-427	HPS27	Intravenous	No carrier second-generation ASO	Metastatic Castrate-Resistant Prostate Cancer	Phase II	NCT01681433
**(b)**						
**Name**	**Target**	**Administration**	**Delivery Method**	**Type of Cancer**	**Phase**	**Clinical Trials ID**
INT-1B3	*miR-193a-3p*	Intravenous	LNP- encapsulated miRNA	Advanced solid neoplasms	Phase I	NCT04675996
*mRNA-5671*	*mRNA-5671* (*KRAS* gene driver mutations)	Intramuscular	LNP- encapsulated miRNA	NSCLC, pancreatic, and colorectal neoplasms	Phase I	NCT03948763
*mRNA-2416*	mRNA Encoding Human OX40L	Intratumoral	LNP- encapsulated miRNA	Advanced malignancies	Phase I/II	NCT03323398
*mRNA-2752*	OX40L, IL-23, IL-36γ	Intratumoral	LNP	Solid tumor or lymphoma	Phase I	NCT03739931
*mRNA 2416*	OX40L	Intratumoral	LNP	Solid tumor or lymphoma	Phase I/II	NCT03323398
BNT116	Activation of cytotoxic T-cells	Intravenous	LNP- mRNA vaccine	NSCLC	Phase I	NCT05142189
Autogene cevumeran	Stimulation of T-cell responses against up to 20 neoantigens	Intravenous	LNP-mRNA vaccine	Resected PDAC	Phase II	NCT05968326
*mRNA-4157 *(V940) Intismeran autogene)	Stimulation of T-cell responses	Intramuscular	LNP-mRNA vaccine	Resected solid tumors	Phase III	NCT05933577, NCT06077760, NCT06295809
RO7198457 (BNT122)	Stimulation of T-cell responses	Intravenous	LNP- mRNA vaccine	CRC	Phase II	NCT04486378
BNT111	NY-ESO-1, tyrosinase, MAGE-A3, TPTE	Intravenous	LNP- mRNA vaccine	Advanced melanoma	Phase I	NCT02410733
BI 1361849 (CV9202)	MAGE-C1, MAGE-C2, NY-ESO-1, survivin, 5T4, MUC1	Intradermal	RNA-lipoflex	Metastatic NSCLC	Phase I/II	NCT03164772

LNP = lipid nanoparticle, PDAC = pancreatic ductal adenocarcinoma.

**Table 9:** Selected examples of clinical trials using liposome/lipoplex-based RNA delivery systems.

Name	Target	Administration	Delivery Method	Type of Cancer	Phase	Clinical Trials ID
MRX34 (*miR-34a* mimic)	MET, MYC, PGDFR-a, CDK4/6, BCL2, PD-L1	Intravenous & Subcutaneous	Liposomal miRNA	Solid tumors, melanoma tumors	Phase I/II	NCT01829971, NCT02862145
Atu027	PKN3	Intravenous	Lipoplex-based siRNA	Pancreatic cancer	Phase I/II	NCT01808638
EphA2 siRNA	EphA2	Intravenous	Liposomal siRNA	Advanced/recurrent solid tumors	Phase I	NCT01591356
Grb2 ASO (BP1001)	Growth factor receptor-bound protein 2	Intravenous	Liposomal ASO	Advanced/recurrent solid tumors	Phase I	NCT04196257
c-Raf ASO	RAF1 (c-Raf kinase)	Intravenous	Liposomal ASO	Advanced solid tumors	Phase I	NCT00024661

PD-L1 = programmed cell death ligand 1, BCL2 = B-cell lymphoma 2.

**Table 10:** Selected examples of clinical trials using extracellular vesicle-based RNA delivery systems.

Name	Target	Administration	Delivery Method	Type of Cancer	Phase	Clinical Trials ID
ASO-STAT6	STAT6 pathway, tumor-immune evasion	Intravenous	Exosome-based ASO	Advanced HCC	Phase I	NCT05375604
IGF-1R/AS-ODN	Insulin-like growth factor type 1 receptor	Intravenous	Exosome-based antisense oligo	Recurrent malignant glioma	Phase I	NCT01550523
Antisense102	Shut down a targeted surface receptor protein, leading to apoptosis	Intravenous	Exosome-based antisense oligo	Newly diagnosed malignant glioma	Phase I	NCT02507583
iExosome or KRAS G12D siRNA	KRAS	Intravenous	Exosome-based siRNA	Pancreatic cancer	Phase I	NCT03608631

**Table 11:** Selected examples of clinical trials using polymer-based RNA delivery systems.

Name	Target	Administration	Delivery Method	Type of Cancer	Phase	Clinical Trials ID
CALAA-01	M2 subunit of ribonucleotide reductase (R2)	Intravenous	Cyclodextrin-based polymer nanoparticle carrying siRNA	Solid tumor	Phase I	NCT00689065
siG12D LODER	KRAS	Local Release	Biodegradable polymer implant delivering siRNA	Locally advanced pancreatic cancer	Phase II	NCT01188785, NCT01676259

**Table 12:** Selected examples of other RNA delivery and targeting approaches.

Name	Type of Delivery Vector	Target	Administration	Delivery Technology	Type of Cancer	Phase	Clinical Trials ID
WT1 mRNA-electroporated dendritic cells	Cell vaccine	Wilms’ tumor 1 protein	Intradermal	Autologous mRNA-electroporated dendritic cells	Acute myeloid leukaemia	Phase I/II	NCT01686334
PERCELLVAC3	Cell vaccine	Tumor-associated antigen mRNAs	Intravenous	Autologous mRNA-loaded dendritic cells	Brain metastases from Solid Tumors	Phase I	NCT02808416
DC/CIK	Cell immunotherapy	SOCS1, MUC1 and Survivin	Intravenous	Dendritic cell/cytokine-induced killer (DC-CIK)	Advanced NSCLC with bone metastases	Phase I/II	NCT02688686
TBI-1301	Retroviral Vector	T-cells	Intravenous	Retroviral engineered T-cells	Refractory solid tumors	Phase I/II	NCT03240861, NCT03250325
LNA-i-miR-221	LNA	*miR-221*	Intravenous	LNA antimiR	Advanced solid neoplasms	Phase I	NCT04811898
EZN-2968	LNA	*HIF-1α* mRNA	Intravenous	LNA antisense oligonucleotide	Advanced solid neoplasms and lymphoma	Phase I	NCT00466583
NOX-A12	RNA aptamer	CXCL12 chemokine binding	Intravenous	RNA aptamer	Relapsed chronic lymphocytic leukemia	Phase I/II	NCT04121455, NCT04901741
NU-0129	Gold nanoparticle	Bcl2L12	Intravenous	Gold nanoparticle	Glioblastoma	Phase I	NCT03020017

LNA = locked nucleic acid, WT1 = Wilms’ Tumor 1 protein, Bcl = B-cell lymphoma.

## Data Availability

No new data were created or analyzed in this study. Data sharing is not applicable to this article.

## Abbreviations

The following abbreviations are used in this manuscript:

5-FU	5-fluorouracil
ACT	adoptive cell therapy
AGO2	argonaute RISC catalytic component 2
APC	antigen-presenting cell
ApoE	apolipoprotein E
ASGR	asialoglycoprotein receptor
ASO	antisense oligonucleotide
ATG3	Autophagy-related 3
AUNP	gold nanoparticle
BC	bladder cancer
BCL2	B-cell lymphoma 2
BCL-XL	B-cell lymphoma extra-large
BRCA	breast cancer
CAF	cancer-associated fibroblast
cAMP	cyclic AMP
CAR-T	chimeric antigen receptor T-cell
CDX	cell line-derived xenograft
ceRNA	competing endogenous RNA
CLL	chronic lymphocytic leukemia
c-MET	c-mesenchymal–epithelial transition
CRC	colorectal cancer
CRISPRi	CRISPR interference
CTCL	cutaneous T-cell lymphoma
CXCL12	C-X-C motif chemokine ligand 12
DDR	DNA damage response
DECR1	2,4-dienoyl-CoA reductase 1
DNA-PKcs	DNA-dependent protein kinase catalytic subunit
DNMT	DNA methyltransferase
DOK1	downstream of tyrosine kinase 1
DOPC	1,2-dioleoyl-sn-glycero-3-phosphatidylcholine
DOTAP	1,2-dioleoyl-3-dimethylammonium-propane
dsRNA	double-stranded RNA
EGFR	epidermal growth factor receptor
EMA	European Medicines Agency
EMT	Epithelial-to-mesenchymal transition
EV	extracellular vesicles
FAO	fatty acid oxidation
FDA	Food and Drug Administration
GalNAc	N-acetylgalactosamine
GC	gastric cancer
GPCR	G-protein-coupled receptor
GPCR-Gαi	G protein-coupled receptor-inhibitory alpha (α) subunit of the G protein
GSH	glutathione
HCC	hepatocellular carcinoma
HER2	human epidermal growth factor receptor 2
HGF	hepatocyte growth factor
HIF-1α	hypoxia-inducible factor 1 alpha
HMGA1	high mobility group A1
HNSCC	head and neck squamous cell carcinoma
Hsp27	heat shock protein 27
hTR	Human telomerase RNA
HuR	Hu antigen R
ICI	immune checkpoint inhibitor
IFN	type I interferon
KIRC	kidney renal clear cell carcinoma
LIHC	liver hepatocellular carcinoma
LNA	locked nucleic acid
lncRNA	long non-coding RNA
LNP	lipid nanoparticle
m6A	N^6^-methyladenosine
mAb	monoclonal antibody
MCM3	Minichromosome Maintenance Complex 3
MDM2	mouse double minute 2 homolog
METTL3	methyltransferase-like 3
MHC-I	major histocompatibility complex class I
miRNA	microRNA
MMP	matrix metalloproteinase
mRNA	messenger RNA
ncRNA	non-coding RNA
NHEJ	non-homologous end joining
N-HER2	nuclear human epidermal growth factor receptor 2
NPC	nasopharyngeal carcinoma
NSCLC	non-small-cell lung cancer
OE	Overexpressed
PAK5	p21-activated kinase 5
PCBP1	Poly(rC)-binding protein 1
PD-1	programmed cell death protein 1
PDAC	pancreatic ductal adenocarcinoma
PD-L1/2	programmed cell death ligand 1 or 2
PDX	patient-derived xenograft
PEG	polyethylene glycol
PEI	Polyethylenimine
PHD2	prolyl hydroxylase domain protein 2
PIP3	phosphatidylinositol-(3,4,5)-triphosphate
PKA	protein kinase A
PKN3	protein kinase N3
PLC	Peptide-Loading Components (not to be mistaken with phospholipase C)
PLGA	poly(lactide-co-glycolactide)
PPARα	peroxisome proliferator-activated receptor alpha
PUFA	polyunsaturated fatty acid
RISC	RNA-induced silencing complex
ROS	reactive oxygen species
SELEX	systemic evolution of ligands by exponential enrichment
siRNA	small interfering RNA
SMKI	small molecule kinase inhibitor
SORT	selective organ-targeting
ssDNA	single-stranded DNA
ssRNA	single-stranded RNA
TAM	tumor-associated macrophage
TCR	T-cell receptor
TLR	Toll-like receptor
TNBC	triple negative breast cancer
TNS4	tension focal adhesion 4
TTR	transthyretin
UE	Underexpressed
WT1	Wilms’ Tumor 1 protein
YTHDC1	YTH domain-containing protein 1
YTHDF3	YTH N^6^-methyladenosine RNA-binding protein 3
